# Identification of QTLs affecting scopolin and scopoletin biosynthesis in *Arabidopsis thaliana*

**DOI:** 10.1186/s12870-014-0280-9

**Published:** 2014-10-18

**Authors:** Joanna Siwinska, Leszek Kadzinski, Rafal Banasiuk, Anna Gwizdek-Wisniewska, Alexandre Olry, Bogdan Banecki, Ewa Lojkowska, Anna Ihnatowicz

**Affiliations:** Intercollegiate Faculty of Biotechnology of University of Gdansk and Medical University of Gdansk, ul. Kladki 24, Gdansk, 80-822 Poland; Université de Lorraine, UMR 1121 Laboratoire Agronomie et Environnement Nancy-Colmar, 2 avenue de la forêt de Haye, Vandœuvre-lès-Nancy, 54505 France; INRA, UMR 1121 Laboratoire Agronomie et Environnement Nancy-Colmar, 2 avenue de la forêt de Haye, Vandœuvre-lès-Nancy, 54505 France

**Keywords:** Coumarins, Natural variation, Plant-environment interaction, Scopoletin, Scopolin, Secondary metabolism, QTL mapping

## Abstract

**Background:**

Scopoletin and its glucoside scopolin are important secondary metabolites synthesized in plants as a defense mechanism against various environmental stresses. They belong to coumarins, a class of phytochemicals with significant biological activities that is widely used in medical application and cosmetics industry. Although numerous studies showed that a variety of coumarins occurs naturally in several plant species, the details of coumarins biosynthesis and its regulation is not well understood. It was shown previously that coumarins (predominantly scopolin and scopoletin) occur in *Arabidopsis thaliana* (Arabidopsis) roots, but until now nothing is known about natural variation of their accumulation in this model plant. Therefore, the genetic architecture of coumarins biosynthesis in Arabidopsis has not been studied before.

**Results:**

Here, the variation in scopolin and scopoletin content was assessed by comparing seven Arabidopsis accessions. Subsequently, a quantitative trait locus (QTL) mapping was performed with an Advanced Intercross Recombinant Inbred Lines (AI-RILs) mapping population EstC (Est-1 × Col). In order to reveal the genetic basis of both scopolin and scopoletin biosynthesis, two sets of methanol extracts were made from Arabidopsis roots and one set was additionally subjected to enzymatic hydrolysis *prior to* quantification done by high-performance liquid chromatography (HPLC). We identified one QTL for scopolin and five QTLs for scopoletin accumulation. The identified QTLs explained 13.86% and 37.60% of the observed phenotypic variation in scopolin and scopoletin content, respectively. *In silico* analysis of genes located in the associated QTL intervals identified a number of possible candidate genes involved in coumarins biosynthesis.

**Conclusions:**

Together, our results demonstrate for the first time that Arabidopsis is an excellent model for studying the genetic and molecular basis of natural variation in coumarins biosynthesis in plants. It additionally provides a basis for fine mapping and cloning of the genes involved in scopolin and scopoletin biosynthesis. Importantly, we have identified new loci for this biosynthetic process.

**Electronic supplementary material:**

The online version of this article (doi:10.1186/s12870-014-0280-9) contains supplementary material, which is available to authorized users.

## Background

Plants produce a great variety of secondary metabolites. It is estimated that between 4000 to 20 000 metabolites per species can be expected [1]. This great biochemical diversity reflects the variety of environments in which plants live, and the way they have to deal with different environmental stimuli. The production of specialized secondary metabolites is assumed to protect plants against biotic and abiotic stresses [2]. Although Arabidopsis is a small plant with short generation time and highly reduced genome, it has a set of secondary metabolites that is as abundant and diverse as those of other plant taxa [3]. In recent years, this model plant was extensively used towards identification of genes and enzymes working in a complex network involved in secondary metabolites biosynthesis and regulation [4].

Currently, genetic variation found between natural Arabidopsis accessions is an important basic resource for plant biology [5-7]. Arabidopsis with its extensive genetic natural variation provides an excellent model to study variation in the biosynthesis of secondary metabolites in natural populations. Recent genetic analysis of natural variation in untargeted metabolic composition uncovered many qualitative and quantitative differences in metabolite accumulation between Arabidopsis accessions [8-10]. Numerous studies [8,10-12] proved the presence of abundant genetically controlled variation for various classes of secondary metabolites. Coumarins (scopoletin, scopolin, skimmin and esculetin) are one of the secondary metabolite classes found in Arabidopsis’ roots [13-16]. But up to now, nothing is known about natural variation in coumarins content between Arabidopsis accessions.

Coumarins are a group of important natural compounds that provide for the plant antimicrobial and antioxidative activities, and are produced as a defence mechanism against pathogen attack and abiotic stresses [17]. Importantly, coumarins are widely recognized in the pharmaceutical industry for their wide range of therapeutic activities and are an active source for drug development. Numerous coumarins have medical application in the treatment of burns and rheumatoid diseases. Furanocoumarins, which are coumarin derivatives, are used in the treatment of leucoderma, vitiligo and psoriasis [18], due to their photoreactive properties. Moreover, they are used in symptomatic treatment of demyelinating diseases, particularly multiple sclerosis [19]. Furanocoumarin-producing plants that are currently studied are non-model organisms [20] and many approaches to identify the genes underlying genetic variation in coumarins accumulation are not yet available in those species. Scopoletin, which is a major coumarin compound of Arabidopsis, has been found in many plant species [21-29], and was clearly shown to have antifungal and antibacterial activities important for medical purposes [30]. All these properties make coumarins attractive from the commercial point of view.

**Figure 1:**
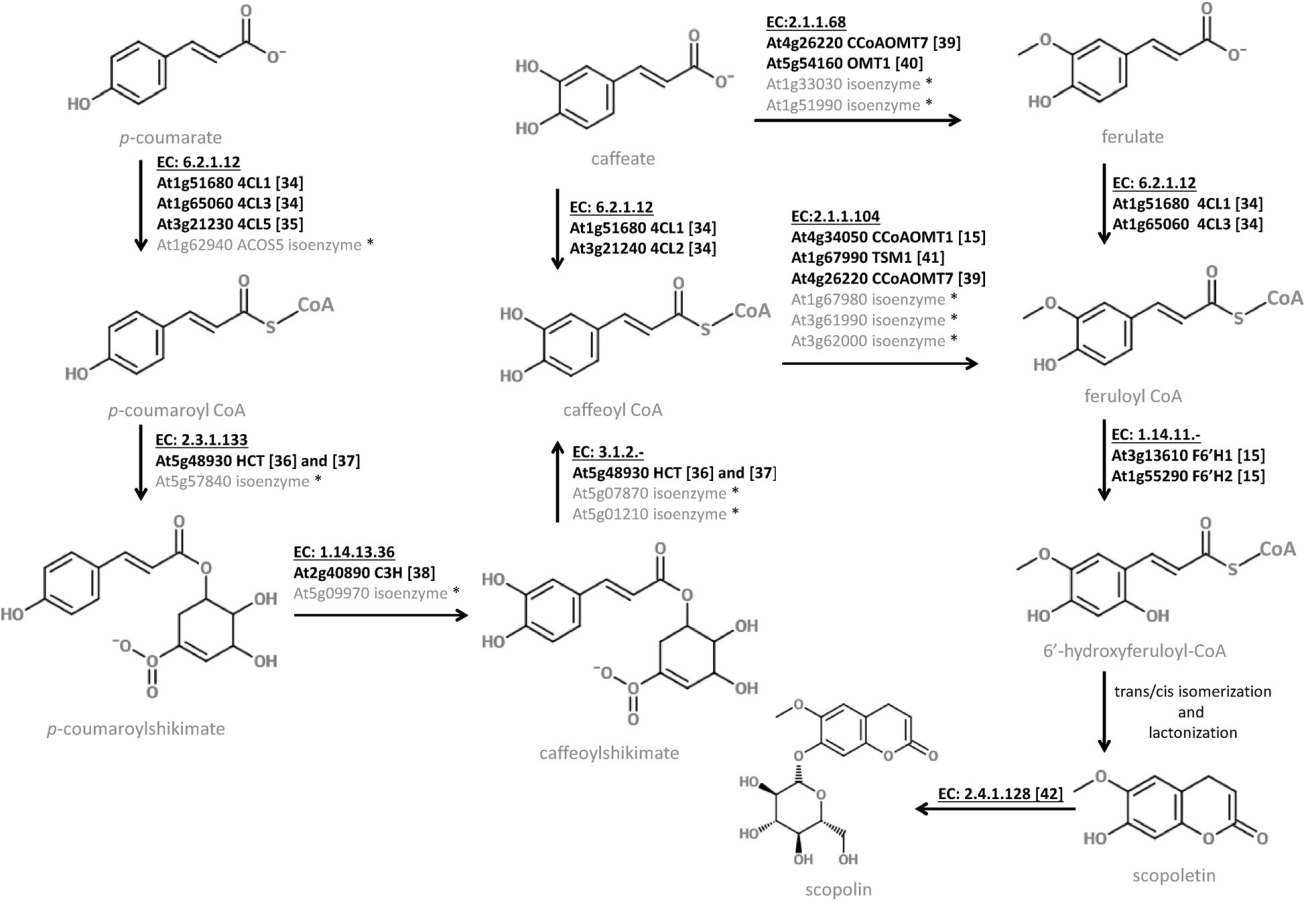
**The chemical structures of scopoletin and scopolin and their biosynthetic pathway in** ***Arabidopsis thaliana*** **.** Cloned and characterized genes encoding enzymes for scopoletin and scopolin biosynthesis are shown. The corresponding references [15,34-42] are listed in the Reference section. The isoenzymes indicated by an asterisk (*) were functionally annotated by the in-house Ensemble Enzyme Prediction Pipeline (E2P2, version 2.0) (Plant Metabolic Network, http://pmn.plantcyc.org/ARA). The presented molecules were created using https://www.emolecules.com/ website. (4CL1) 4-coumarate:CoA ligase 1. (4CL2) 4-coumarate:CoA ligase 2. (4CL3) 4-coumarate:CoA ligase 3. (4CL5) 4-coumarate:CoA ligase 5. (C3H) p-coumaroyl 3′-hydroxylase. (CCoAOMT1) caffeoyl coenzyme A dependent O-methyltransferase 1. (CCoAOMT7) caffeoyl coenzyme A dependent O-methyltransferase 7. (F6′H1) feruloyl-CoA 6′-hydroxylase 1. (F6′H2) feruloyl-CoA 6′-hydroxylase 2. (HCT) shikimate O-hydroxycinnamoyltransferase. (OMT1) caffeate O-methyltransferase 1. (TSM1) tapetum-specific O- methyltransferase.

Coumarins are derived from phenylopropanoid pathway, which serves as a rich source of metabolites in plants [31,32]. It was suggested that in Arabidopsis several branch pathways leading from phenylpropanoid compounds to coumarins are probable [14]. Scopoletin and scopolin biosynthesis was shown to be strongly dependent on the CYP98A3 [14], which is the cytochrome P450 catalyzing 3′-hydroxylation of p-coumarate units in the phenylpropanoid pathway [33]. The feruloyl-CoA was suggested to be a major precursor in scopoletin biosynthesis [15]. A key enzyme involved in the final step of scopoletin biosynthesis, which is the conversion of feruloyl-CoA into 2-hydroxy-feruloyl-CoA, is encoded by a member of the iron (Fe) II- and 2-oxoglutarate-dependent dioxygenase (2OGD) family, designated as F6′H1 [15]. Despite the advances that have been made in previous years [15,34-42] (Figure 1), many questions with regard to coumarins biosynthesis are still open [43]. In particular, the regulation of the biosynthesis of coumarins is not well understood. Up to now, all studies investigating coumarins biosynthesis in the model plant Arabidopsis were done with one laboratory accession Col-0, which was used as the genetic background of all mutant and transgenic plants.

To gain an understanding of the genetic architecture of coumarins biosynthesis, we screened a set of Arabidopsis accessions for variation in scopolin and scopoletin content, and subsequently conducted a quantitative trait locus (QTL) mapping. Our study addressed the following questions. Is there a natural variation in accumulation of scopolin and scopoletin between Arabidopsis accessions and what are genetic regions responsible for the observed differences? What are candidate genes possibly underlying QTLs involved in scopolin and scopoletin biosynthesis?

## Results

### Phenotypic variation between accessions

**Figure 2:**
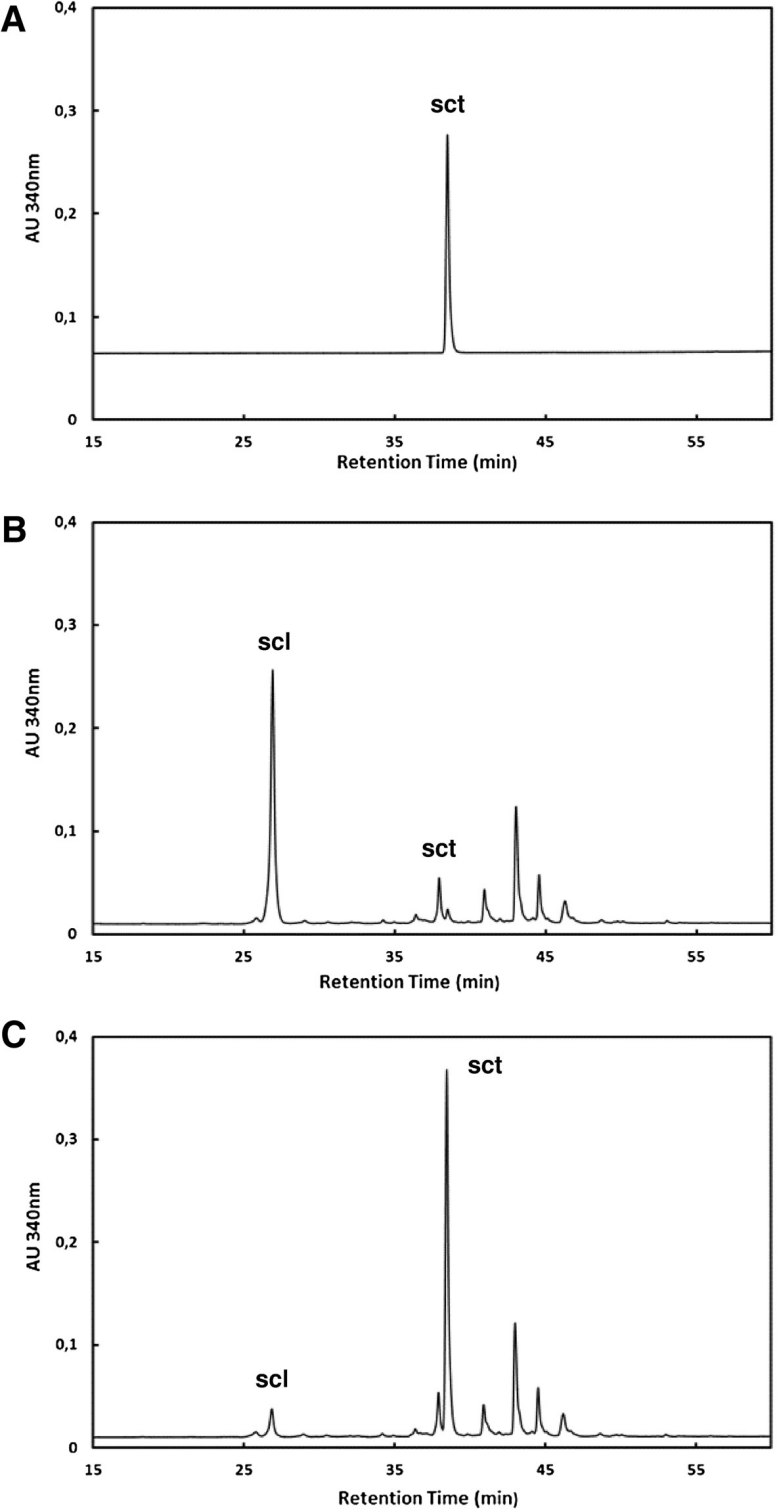
**Chromatograms of scopoletin standard and methanol extracts from** ***Arabidopsis thaliana*** **roots.** The column effluent was monitored with fluorescence detector with excitation at 340 nm and emission at 460 nm. The peak for glucoside of scopoletin – scopolin (scl); the peak for scopoletin (sct). **(A)** Chromatogram of scopoletin standard. **(B)** Chromatogram of methanol extract from Arabidopsis roots before enzymatic hydrolysis. **(C)** Chromatogram of methanol root extract subjected to hydrolysis using β-glucosidase. The peak for scopoletin is a dominant peak of total chromatogram.

**Figure 3:**
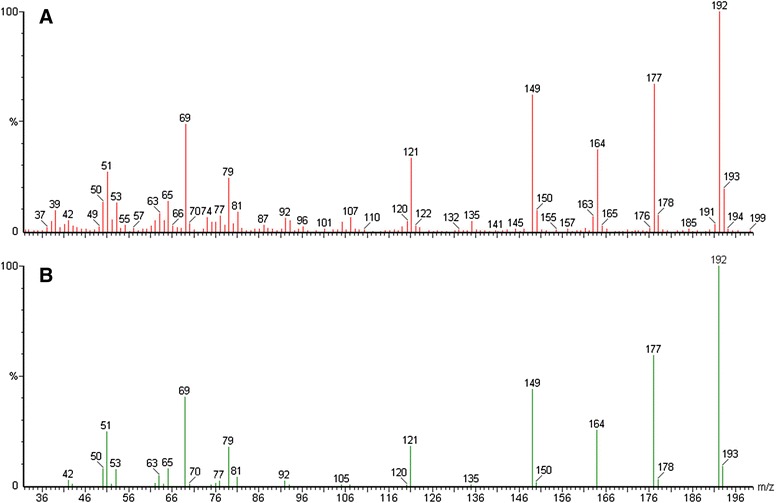
**Mass spectra of HPLC scopoletin fraction and scopoletin standard. (A)** GC/MS spectrum of the scopoletin fraction of methanol extract from *Arabidopsis thaliana* roots subjected to enzymatic hydrolysis. **(B)** Scopoletin standard library spectrum.

**Figure 4:**
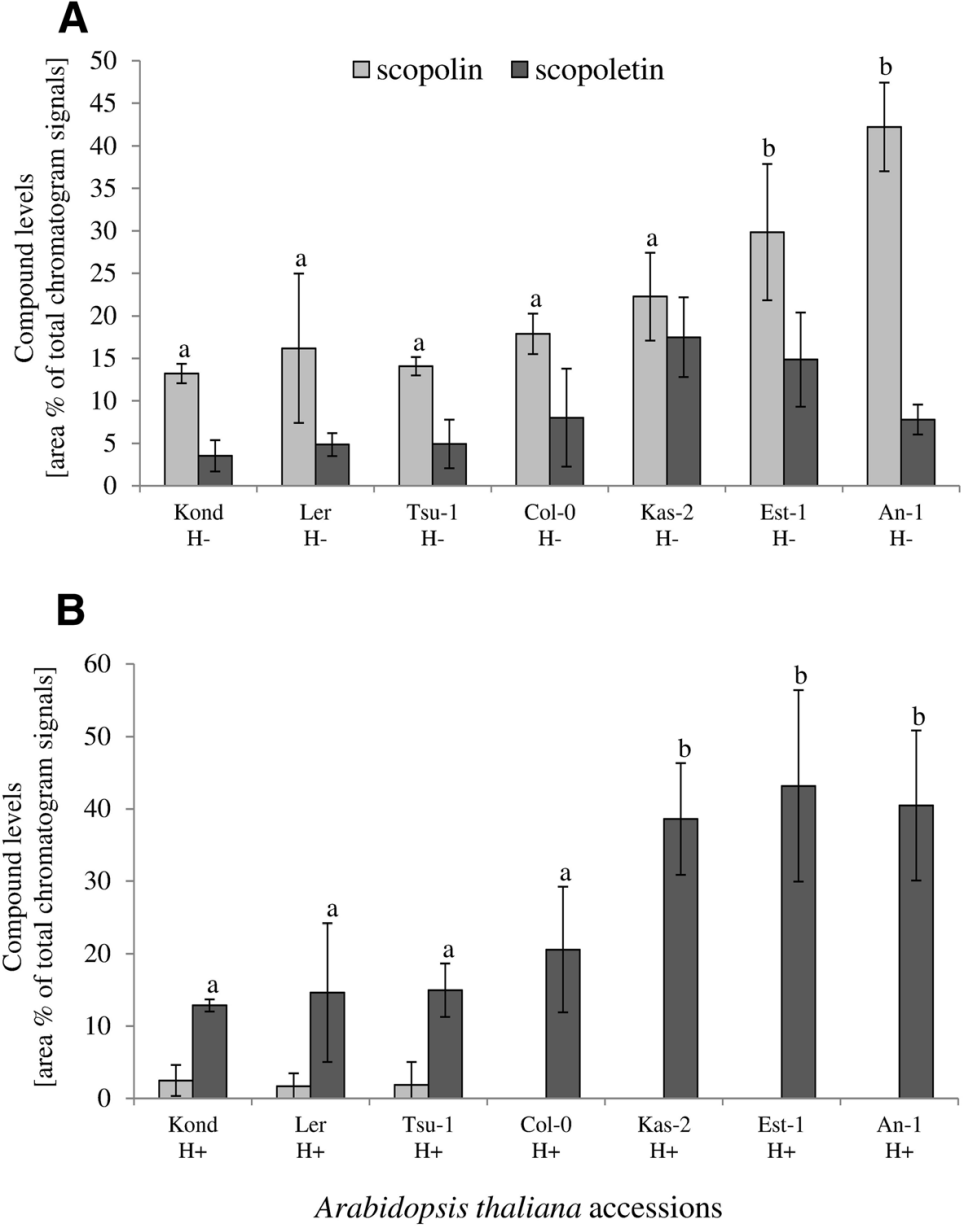
**Relative levels of scopolin and scopoletin in the roots of seven** ***Arabidopsis thaliana*** **accessions. (A)** Scopolin level in methanol root extracts without hydrolysis (H-). **(B)** Scopoletin content in the methanol extracts that were subjected to enzymatic hydrolysis (H+) *prior to* quantification. The statistically significant differences between group means for scopolin and scopoletin accumulation were determined by one-way ANOVA (p < 0.001 and p < 0.0001, respectively). Values that are not significantly different based on the post hoc test (least significant differences [LSD]) are indicated by the same letters. The data analysis consisted of scopolin and scopoletin relative levels measured as area% of total chromatogram signals. Error bars represent the SD from three measurements.

A set of seven natural Arabidopsis accessions, which are the parents of existing RIL populations and represent accessions from different locations, were used in the initial screening for variation in scopolin and scopoletin accumulation. Accessions were grown *in vitro* in liquid cultures in order to obtain the optimal growth of plant roots. Under these conditions, most of the scopoletin is stored in root cells in vacuoles as its glycoside form, scopolin. In order to reveal the content of both scopolin and that of scopoletin, a subset of the methanol extracts made from Arabidopsis roots were subjected to enzymatic hydrolysis in order to hydrolyze the glycoside forms of coumarins. Using high-performance liquid chromatography (HPLC), we detected in the roots scopoletin (sct in Figure 2), as well as scopolin (scl in Figure 2BC). The identification of scopoletin in HPLC fraction (Figure 3A) was further confirmed using gas chromatography/mass spectrometry (GC/MS) by comparison to spectrum library (Figure 3B). The quantification of coumarins in methanol root extracts made from seven Arabidopsis accessions clearly showed the presence of natural variation in scopolin content before enzymatic hydrolysis (Figure 4A) and scopoletin after hydrolysis (Figure 4B). In spite of the fact that scopolin standard was not available and in order to unify further analysis, we measured the amounts of both scopolin and scopoletin as area% of total chromatogram signals. The statistically significant differences between group means for scopolin and scopoletin accumulation were determined by one-way ANOVA (p < 0.001 and p < 0.0001, respectively). Values that are not significantly different based on the post hoc test (least significant differences [LSD]) are indicated by the same letters (Figure 4). Based on the obtained results we have selected an Advanced Intercross Recombinant Inbred Lines (AI-RILs) mapping population derived from the cross between Col-0 and Est-1, because these parents significantly differed in coumarins content. Further genetic analysis was performed using values for the accumulation of scopolin before enzymatic hydrolysis and the content of scopoletin after hydrolysis of methanol extracts.

### Genetic analyses of scopolin and scopoletin accumulation

**Figure 5:**
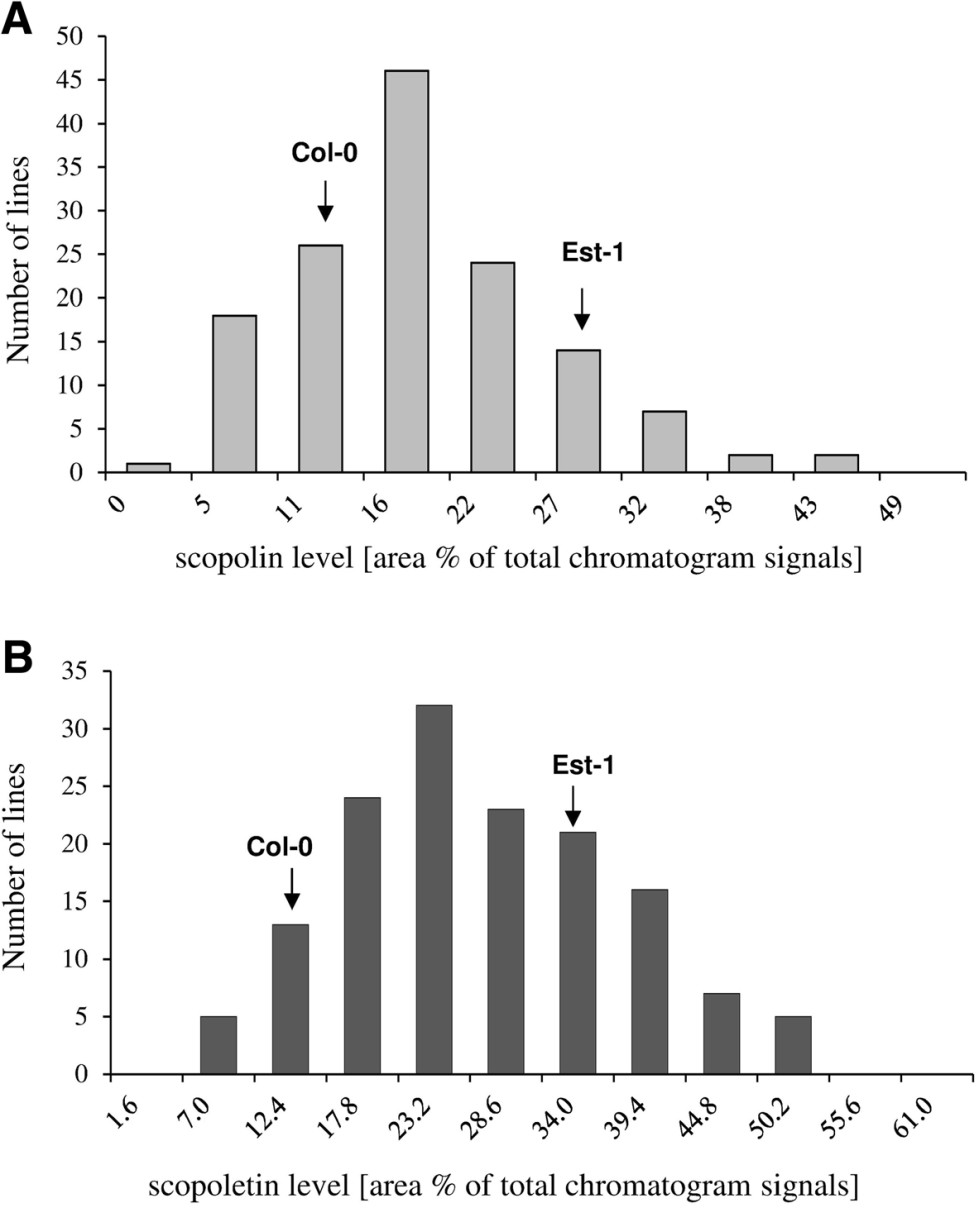
**Frequency distribution of scopolin and scopoletin relative levels in the AI-RILs and parental lines roots.** Plants used for genetic mapping were grown in *in vitro* liquid cultures under a photoperiod of 16 h light (35 μmol m^−2^ s^−1^) at 20°C and 8 h dark at 18°C. Coumarins content in the roots of the AI-RILs population and parental lines (accessions Col-0 and Est-1) were determined by HPLC. **(A)** Scopolin contents were determined in methanol extracts without hydrolysis. **(B)** Methanol extracts subjected to enzymatic hydrolysis were used for scopoletin quantification. The data analysis consisted of scopolin and scopoletin relative levels measured as area% of total chromatogram signals. The average values of Col-0 and Est-1 are indicated with arrows.

The scopoletin and scopolin content values were determined for three biological replicates of AI-RILs (n = 144 and n = 140, respectively) and parental lines, which were grown in independent flasks in liquid cultures. A set of lines (AI-RILs) showed a wider range of scopolin (Figure 5A) and scopoletin (Figure 5B) values than the ones observed for both parental lines (Col-0 and Est-1), which indicated the presence of transgressive segregation and suggested that multiple loci contribute to variation in the EstC population. The lowest scopolin content within AI-RILs was 1.90 (measured as an area% of total chromatogram signals) that corresponds to 20% of the minimum Col-0 value. The maximal relative value of scopolin was 45.13, which corresponds to 159% of the maximal Est-1 value. For scopoletin content, these values were respectively 7.82 (54% of the minimum Col-0 value) and 54.93 (159% of the maximal Est-1 value) (Table 1). Having a commercially available scopoletin standard, we were able to quantify the scopoletin contents as μg/g fresh weight (μg/gFW) in both parental lines of the AI-RILs mapping population (Col-0 and Est-1) before and after enzymatic hydrolysis. The scopoletin levels in root samples not subjected to hydrolysis were ~3 μg/gFW and ~10 μg/gFW in Col-0 and Est-1 respectively, and ~16 μg/gFW and ~86 μg/gFW in samples after hydrolysis. These values correspond to ~18, 54, 82 and 449 nmol/gFW respectively that is in the range found in the literature data, which vary from ~1 to 1200 nmol/gFW depending on plant culture being used [14]. The calculated quantities of parental lines (Table 2) can be used as references for the overall quantity of the products in the whole mapping population.

**Table 1:** **Parental values, ranges and heritabilities in the AI-RILs of scopolin and scopoletin content (relative values** ^**a**^ **)**

	**Parents**		**AI-RIL**		
**Trait**	**Col-0 value** ^**a**^	**Est-1 value** ^**a**^	**Range**	**Mean**	**Heritability** ^**d**^
**Scopolin (H-)** ^**b**^	9.71	28.45	1.9-45.13	19.84	0.50
**Scopoletin (H+)** ^**c**^	14.58	34.53	7.82-54.93	29.68	0.45

^a^Relative levels measured as an area% of total chromatogram signals (as described in Methods section).

^b^Content of scopolin before enzymatic hydrolysis.

^c^Content of scopoletin after enzymatic hydrolysis.

^d^Measure of total phenotypic variance attributable to genetic differences among genotypes (broad sense heritability) calculated as *V*_*G*_ */(V*_*G*_ *+ V*_*E*_ *).*

**Table 2:** **The quantified levels of scopoletin** ^**a**^ **in** ***Arabidopsis thaliana*** **roots**

	**Roots (μg/gFW)**		**Roots (nmol/gFW)**	
	**Col-0 value** ^**a**^	**Est-1 value** ^**a**^	**Col-0 value** ^**a**^	**Est-1 value** ^**a**^
**Scopoletin (H-)** ^**b**^	3.4 ± 1.8	10.4 ± 2.4	17.7 ± 9.4	54.1 ± 6.2
**Scopoletin (H+)** ^**c**^	15.8 ± 6.4	86.2 ± 9.8	82.2 ± 33.3	448.6 ± 51.0

^a^Scopoletin was quantified with HPLC. Values show the averages of scopoletin contents with standard deviation (SD) from two measurements.

^b^Content of scopoletin before enzymatic hydrolysis.

^c^Content of scopoletin after enzymatic hydrolysis.

**Figure 6:**
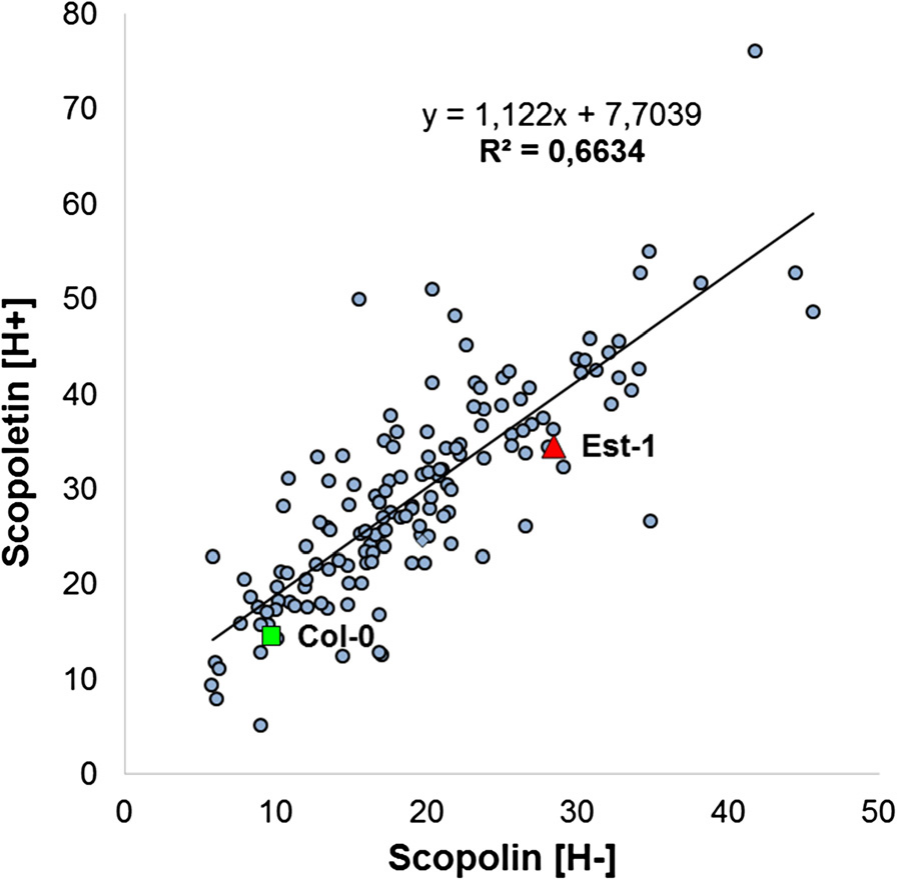
**Scatterplot for scopolin (H-) versus scopoletin (H+) content in the AI-RILs population.** Correlation between scopolin level determined in the methanol root extracts before enzymatic hydrolysis and scopoletin accumulation in extracts subjected to hydrolysis. The regression equation for the AI-RILs population is y = 1.122x +7.7039 with an R^2^ = 0.6634. (□) and (∆) correspond to Col-0 and Est-1 mean values, respectively.

In order to identify the fraction of variation that is genetically determined, the broad sense heritability (*H*^*2*^) for scopolin and scopoletin content was estimated as described in Methods section. In the AI-RIL population, the broad sense heritability ranged from 0.45 for scopoletin to 0.50 for scopolin content (Table 1). To explore the relationship between scopolin content in methanol root extracts before enzymatic hydrolysis and scopoletin levels in extracts subjected to hydrolysis, the mean values of coumarins for each AI-RILs were used as phenotype values in trait correlation analysis. A relatively strong genetic correlation (R^2^ = 0.6634) was observed between the level of coumarins measured before and after hydrolysis in the AI-RILs population, indicating genetic co-regulation of scopolin and scopoletin biosynthesis (Figure 6).

### Mapping QTLs for scopolin and scopoletin accumulation

**Figure 7:**
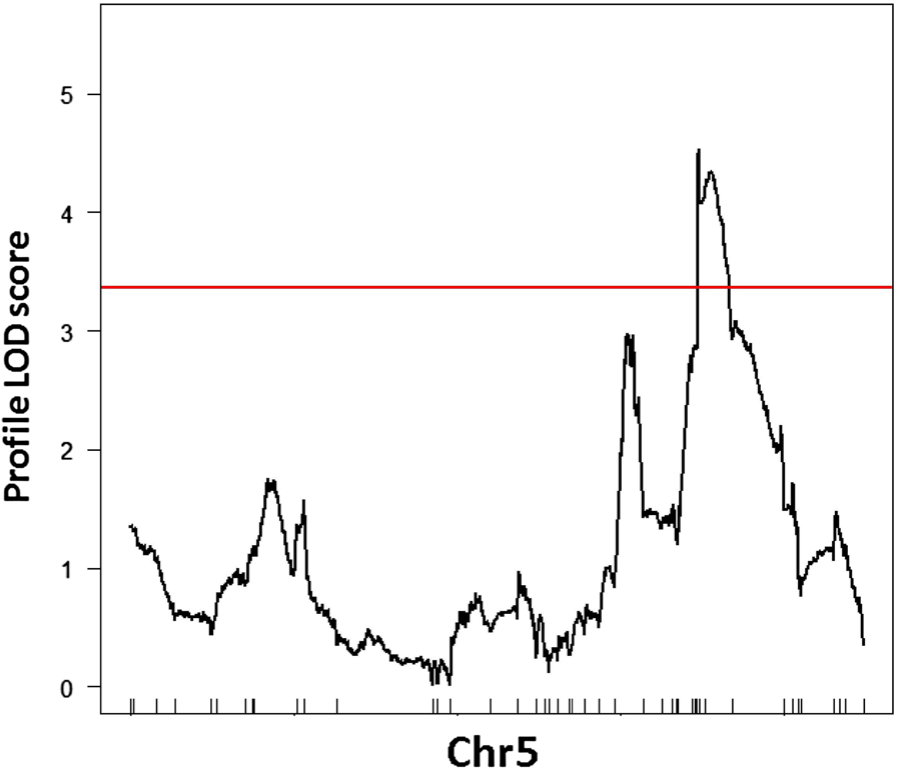
**LOD profile for QTL underlying scopolin accumulation in the AI-RILs.** One-dimensional LOD profile for the QTL underlying variation in scopolin accumulation (SCL1). Red line represents LOD threshold (3.4).

**Figure 8:**
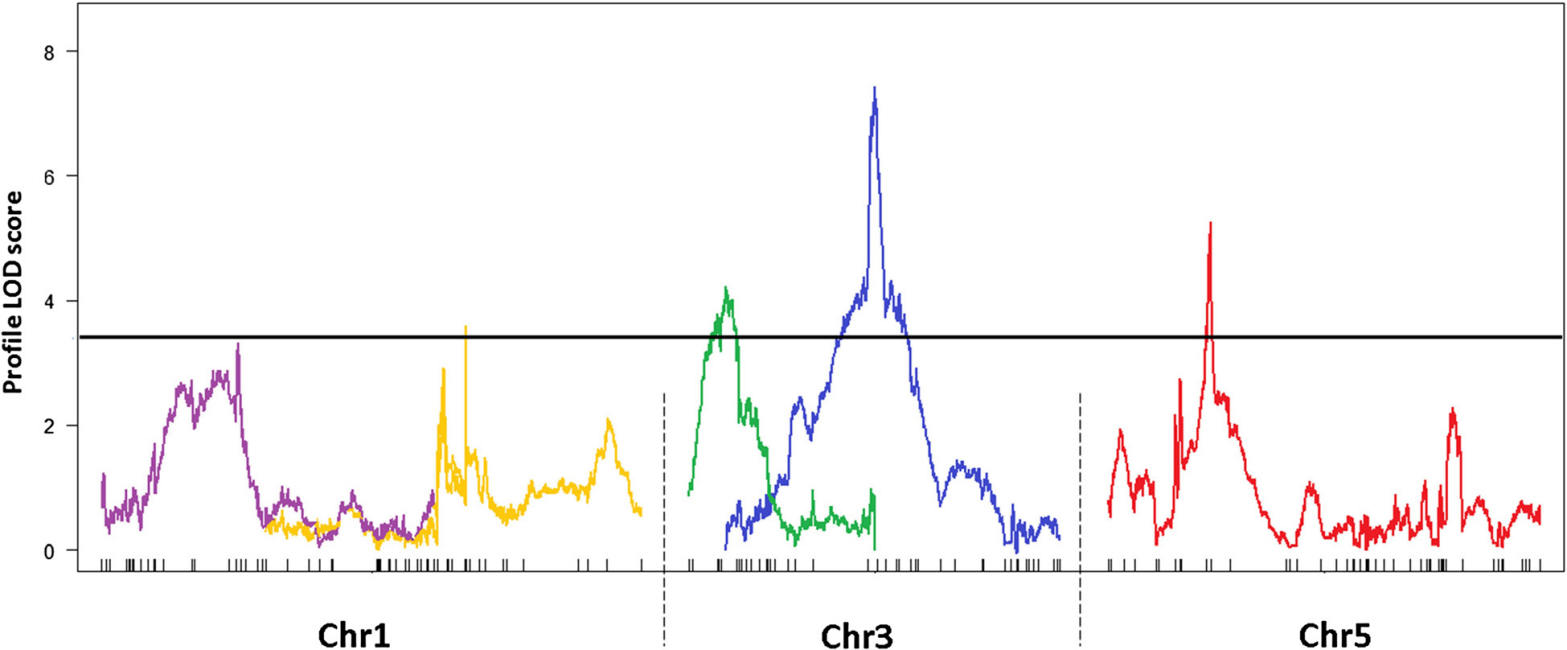
**LOD profiles for QTLs underlying scopoletin accumulation in the AI-RILs.** QTLs for scopoletin (SCT1 - SCT5) content. Black line represents LOD threshold (3.4). Profile LOD curves for a five-QTL model was done with formula = y ~ Q1 + Q2 + Q3 + Q4 + Q5. Each QTL is highlighted in different colour.

Six QTLs were identified, with one QTL being detected for scopolin and five QTLs for scopoletin accumulation (Table 3). The QTL effect sizes ranged from the 7.0% to 16.7% of the phenotypic variance explained by the QTL (PVE), with three of the six QTLs having effect sizes below 10% PVE. One QTL (SCL1) was detected for scopolin accumulation at the bottom of chromosome 5 (Figure 7) explaining the 13.86% PVE (Table 3), and five QTLs (SCT1 - SCT5) for scopoletin accumulation were identified on chromosome 1, 3 and 5 (Figure 8, Table 3). No QTLs were detected on chromosome 2 and 4. To improve the QTL model explaining variation in a scopoletin content, the MQM approach was performed using two QTLs (SCT4 and SCT5) as cofactors. We have included in the model QTL on chromosome 1 (SCT1), despite its LOD score was slightly below the threshold (3.327). The whole model explains 37.6% variance for scopoletin content. No epistasis between the main effect loci were detected.

**Table 3:** **Characteristics of the detected QTLs underlying scopolin and scopoletin biosynthesis in AI-RILs population**

**Trait**	**QTL**	**Chr** ^**a**^	**LOD score**	**Peak** ^**b**^ **(cM)**	**Confidence interval** ^**c**^ **(cM)**	**Confidence interval (bp)**	**PVE** ^**d**^ **(%)**
Scopolin	SCL1	5	4.53	174.2	173.6 - 185.9	19.414.594 - 22.027.830	13.86
Scopoletin	SCT1	1	3.327	71.1	32.6 - 178.6	4.826.763 - 20.083.545	7.008
	SCT2	1	3.594	189.3	176.5 - 263.2	19.672.910 - 28.537.561	7.602
	SCT3	3	4.223	19.2	6.7 - 25.8	786.303 - 4.140.699	9.027
	SCT4	3	7.427	96.7	93.8 - 99.0	9.942.057 - 10.995.480	16.735
	SCT5	5	5.249	53.3	51.7 - 53.9	4.235.132 - 5.725.918	11.409

^a^Chromosome number.

^b^Position of peak.

^c^1-LOD support interval.

^d^Percentage of phenotypic variance explained by the QTL (PVE).

### QTL mapping identifies known and new loci for coumarins biosynthesis

Some of the mapped QTLs underlying variation in scopolin (SCL1) and scopoletin (SCT1 and SCT2) accumulation in the AI-RILs population, co-localize with the genes annotated to be involved in coumarin biosynthetic process (Plant Metabolic Network, http://plantcyc.org/, Figure 1). We detected seven cloned and characterized genes encoding enzymes for scopoletin and scopolin biosynthesis that co-localize with detected QTLs (see Additional file 1). Within the SCL1 interval, which is characterized by one of the highest LOD score values, there are two very good candidates. One of them is At5g48930 encoding a shikimate O-hydroxycinnamoyltransferase (HCT), while the other one (At5g54160) encodes caffeic acid/5-hydroxyferulic acid O-methyltransferase (OMT1). Importantly, both genes are expressed in roots (SCL1 in Table 4). Within the SCT1 and SCT2 intervals underlying variation in scopoletin content more possible candidate genes were detected: At1g33030, At1g51990, At1g67980 and At1g67990 (TSM1) encoding proteins from O-methyltransferase family; At1g51680 and At1g65060 encoding isoforms of 4-coumarate:CoA ligase (4CL1 and 4CL3 respectively); At1g62940 encoding acyl-CoA synthetase (ACOS5); and At1g55290 encoding feruloyl CoA ortho-hydroxylase 2 (F6′H2).

**Table 4:** **Potential candidate genes** ^**a**^ **located within the SCL1, SCT4 and SCT5 intervals**

**No.**	**Locus: Description (** ***TAIR*** **)**	**Gene expression level (** ***Arabidopsis eFP Browser*** **)**	
		**Relative level (Fold-change)**	**Absolute level**
*Candidate genes selected from the QTL interval (SCT4) on chromosome 3: 9942057 to 10995480 nt.*			
1.	AT3G27230: S-adenosyl-L-methionine-dependent methyltransferases superfamily protein	1.18	210.15
2.	AT3G27325: Hydrolases, acting on ester bond	1.20	40.38
3.	AT3G27340: Molecular_function unknown; involved in oxidation reduction	1.62	83.80*
4.	AT3G27890: Encodes NAD(P)H:quinone reductase	1.59	367.30
5.	AT3G28200: Peroxidase superfamily protein	2.42	242.03
6.	AT3G28480: Oxoglutarate/iron-dependent oxygenase	0.82	130.66
7.	AT3G28740: Encodes a member of the cytochrome p450 family (CYP81D11)	3.35	35.84
*Candidate genes selected from the QTL interval (SCT5) on chromosome 5: 4235132 to 5725918 nt.*			
8.	AT5G13780: Acyl-CoA N-acyltransferases (NAT) superfamily protein	1.96	395.68
9.	AT5G14130: Peroxidase superfamily protein	4.02	16.68
10.	AT5G14240: Thioredoxin superfamily protein	2.49	397.36*
11.	AT5G14340: Member of the R2R3 factor gene famil (MYB40)	5.16	28.41*
12.	AT5G14390: Alpha/beta-Hydrolases superfamily protein	1.36	69.43
13.	AT5G14430: S-adenosyl-L-methionine-dependent methyltransferases superfamily protein	1.26	261.78
14.	AT5G14750: Encodes a MyB-related protein containing R2 and R3 repeats (MYB66)	64.45	99.90*
15.	AT5G15130: Encodes a member of WRKY Transcription Factor (WRKY72)	18.65	85.83*
16.	AT5G15180: Peroxidase superfamily protein	36.11	287.14^#^
*Candidate genes selected from the QTL interval (SCL1) on chromosome 5: 19414594 to 22027829 nt.*			
17.	AT5G47950: HXXXD-type acyl-transferase family protein	11.16	68*
18.	AT5G47980: HXXXD-type acyl-transferase family protein	28.79	66*
19.	AT5G47990: Encodes a member of the CYP705A family of cytochrome P450 enzymes	40.50	168*
20.	AT5G48000: Encodes a member of the CYP708A family of cytochrome P450 enzymes	221.82	189*
21.	AT5G48020: 2-oxoglutarate (2OG) and Fe(II)-dependent oxygenase superfamily protein	1.22	177
22.	AT5G48560: Basic helix-loop-helix (bHLH) DNA-binding superfamily protein	11.99	124
23.	AT5G48930: Encode shikimate O-hydroxycinnamoyltransferase (HCT)^b^	1.70	757
24.	AT5G49520: Encodes WRKY48, a member of the WRKY Transcription Factor	3.86	175*
25.	AT5G49560: Putative methyltransferase family protein	2.95	71
26.	AT5G49810: Methionine S-methyltransferase (MMT)	1.27	349
27.	AT5G49950: Alpha/beta-Hydrolases superfamily protein	1.16	119
28.	AT5G50890: Alpha/beta-Hydrolases superfamily protein	1.27	36
29.	AT5G51130: S-adenosyl-L-methionine-dependent methyltransferases superfamily protein	1.41	46.65
30.	AT5G51880: 2-oxoglutarate (2OG) and Fe(II)-dependent oxygenase superfamily protein	0.99	291.46
31.	AT5G52260: Encodes a member of the R2R3 factor gene family (MYB19)	4.83	30.21*
32.	AT5G52400: Encodes a member of CYP715A	1.03	12.91
33.	AT5G53560: Encodes a cytochrome b5 isoform that can be reduced by AtCBR	1.54	1845.23
34.	AT5G53990: UDP-Glycosyltransferase superfamily protein	19.54	10.75^#^
35.	AT5G54080: Homogentisate 1,2-dioxygenase (HGO)	1.53	254.15
36.	AT5G54160: OMT1:A caffeic acid/5-hydroxyferulic acid O-methyltransferase (OMT1)^b^	1.01	758.6
37.	AT5G54230: Encodes a putative transcription factor (MYB49)	7.01	34.73*

^a^The list of potential candidate genes was compiled by searching TAIR (http://www.arabidopsis.org/) and Arabisopsis eFP Browser (http://bar.utoronto.ca/).

^b^Loci known to be involved in coumarins biosynthesis.

^*^Genes with the highest expression in roots of vegetative rosette.

^#^Genes with relatively high expression in roots of vegetative rosette.

The selected intervals are associated with scopolin (SCL1) and scopoletin (SCT4, SCT5) accumulation and are characterized by the highest percentage of phenotypic variance explained by each QTL and the highest LOD score values. Most of selected genes (except two highlighted with letter ^b^) are novel loci.

In order to reveal other candidate genes possibly underlying detected QTLs, two QTLs for scopoletin content (SCT4 and SCT5) and one QTL associated with scopolin (SCL1) accumulation were chosen for further *in silico* analyses. The selected intervals are characterized by the highest percentage of phenotypic variance explained by each QTL and the highest LOD score values. The annotated functions for all genes located in the selected QTL intervals were checked. As a result, we selected genes encoding transcription factors that might be induced by environmental stresses and enzymes that according to the annotation functions could be possibly involved in scopolin and scopoletin biosythensis. Subsequently, we performed *in silico* analysis of the tissue distribution and level of expression of selected genes. Only genes that were expressed in roots were selected as possible candidates for further studies. As a result, we selected a set of genes that deserve close attention as possible new loci underlying variation in scopolin and scopoletin accumulation (Table 4). Among candidates possibly involved in scopoletin accumulation, a particularly interesting one is a *CYP81D11* gene (At3g28740) encoding a member of the cytochrome P450 family, which is located within the QTL on chromosome 3 (SCT4 in Table 4). According to the 1001 Genomes Project database (www.1001genomes.org) and re-sequencing data of Est-1 from our laboratory (see Additional files 2 and 3, indicated as Est-1*), the *CYP81D11* gene contains several SNPs and one indel in the coding sequences of the parental lines of EstC mapping population and in the other accessions tested in this study (see Additional file 2). Other interesting candidates are three genes (At5g14340, At5g14750, At5g15130) located within the QTL interval on chromosome 5 (SCT5 in Table 4), which encode members of the MYB and WRKY transcription factor families. These genes are relatively highly expressed in roots and their expression is induced by various environmental stresses [44]. A particularly interesting candidate that could be possibly linked to scopolin accumulation was detected within the QTL on chromosome 5 (SCL1 in Table 4). It is At5g53990 encoding a UDP-glycosyltransferase, which is relatively highly expressed in Arabidopsis roots [44]. According to the 1001 Genomes Project and our re-sequencing data of Est-1, this gene contains several SNPs in the coding sequences of tested accessions including the parental lines (see Additional file 3). Interestingly, the *CYP81D11* and *UDP-glycosyltransferase* sequences originating from Est, Est-1 (both taken from the 1001 Genomes Project database) and Est-1* that was re-sequenced in our laboratory are not identical (see Additional files 2 and 3). This needs to be further verified.

## Discussion

Here, we report a QTL mapping study of variation in scopoletin and scopolin accumulation between two Arabidopsis accessions and thereby we demonstrate the usefulness of Arabidopsis natural variation in elucidating the genetic and molecular basis of coumarins biosynthesis.

A large number of Arabidopsis recombinant inbred line (RIL) populations are available and extensively used for identification of numerous QTLs controlling various traits such as growth, development or resistance to different biotic and abiotic stresses as well as the content of chemical compounds [5,7,9,45,46]. In most studies, the average number of QTLs identified is between one and 10 and at least one major QTL is detected [47]. Here, one QTL for scopolin and five QTLs for scopoletin accumulation were detected, which is in agreement with the average result in the field. Using an AI-RILs mapping population has the advantage in comparison to RILs due to the fact that the opportunity for recombination is increased before genotypes are fixed upon selfing [48]. As a result, using AI-RILs mapping population that captures an increased number of recombination events [48], enabled us to detect QTLs with effect size as low as 7.0% PVE.

Once QTL has been identified, the next challenge is to identify the gene(s) underlying detected QTL. In most cases, a large number of genes that are present in the QTL interval cannot be directly tested for candidacy. In order to reduce the mapped region, a fine-mapping is performed in which many individuals are genotyped for markers around the QTL. More accurate QTL localization might lead to the selection of candidate genes. Nonetheless, performing a fine mapping may be practically difficult if the QTL effect is relatively small [49]. When multiple data sets are available, which is the case for Arabidopsis, it is possible to improve accuracy and to test the candidacy of genes within mapped QTL intervals [49] based on the available information. Therefore, it seems like a realistic possibility to identify candidate genes underlying a QTL by using the high throughput expression data and the complete genome sequences of numerous Arabidopsis accessions that were used to construct mapping populations. There are successful examples of using expression arrays in identifying genes causally associated with quantitative traits of interest, both in plants and animals [50,51]. In this study, possible candidate genes were found within mapped QTL intervals for scopolin and scopoletin content, including known and novel loci. Further functional analysis, including re-sequencing, characterization of loss-of-function alleles and conducting gene complementation either by crossing or genetic transformation, are required to prove the role of selected possible candidate genes in coumarins biosynthesis and their regulation.

Expanding molecular understanding of coumarins biosynthesis at an ecological level will be beneficial for the future discovery of the physiological mechanisms of action of genes involved in coumarins biosynthesis. It was suggested recently that some members the 2′-OG dioxygenase family, including the F6′H1 that is a key enzyme in scopoletin biosynthesis, may be involved in Fe deficiency responses and metabolic adjustments linked to Fe homeostasis in plant cells [52]. Other latest studies showed that Fe deficiency induces the secretion of scopoletin and its derivatives by Arabidopsis roots [53], and that F6′H1 is required for the biosynthesis of coumarins that are released into the rhizosphere as part of the strategy I-type Fe acquisition machinery [54]. Previously, the existence of natural variation in root exudation profiles was clearly detected among eight Arabidopsis accessions [55]. The above mentioned findings make a study of coumarins biosynthesis in Arabidopsis using naturally occurring intraspecific variation even more promising and up-to-date.

## Conclusions

In summary, we have presented here for the first time a presence of naturally occurring intraspecies variation in scopoletin and its glucoside, scopolin, accumulation among seven Arabidopsis accessions. Even though, these accessions do not completely represent a wide genetic variation existing in Arabidopsis, it is assumed that these accessions should reflect genetic adaptation to local environmental factors [6]. A QTL mapping study of scopoletin and scopolin variation within EstC mapping population was conducted leading to the identification of new loci. The results presented here suggest that natural variation in coumarins content in Arabidopsis has a complex molecular basis. Importantly, they also provide a basis for fine mapping and cloning of the genes involved in coumarins biosynthesis.

## Methods

### Plant material

Seven *Arabidopsis thaliana* accessions Antwerpen (An-1, Belgium), Columbia (Col-0, Germany), Estland (Est-1, Estonia), Kashmir (Kas-2, India), Kondara (Kond, Tadjikistan), *Landsberg erecta* (L*er*, Poland) and Tsu (Tsu-1, Japan), which are the parents of existing RIL populations and represent accessions from different locations, were used in the initial screening for variation in scopoletin and scopolin accumulation. An advanced recombinant inbred lines (AI-RILs) mapping population (EstC) derived from the cross between Columbia (Col-0) and Estland (Est-1) was used in the QTL mapping experiment [48]. All seeds of the Arabidopsis accessions and mapping population were kindly provided by Maarten Koornneef from the Max Planck Institute for Plant Breeding Research in Cologne, Germany. Arabidopsis accessions are available at the stock centre NASC (http://arabidopsis.info/). The EstC mapping population together with the marker data are available at the NASC under the stock number CS39389.

### Growth conditions

The seeds were surface sterilized by soaking in 70% ethanol for two min and subsequently kept in 5% calcium hypochlorite solution for eight min. Afterwards seeds were rinsed three times in autoclaved millipore water and planted on 0.5 Murashige and Skoog’s (MS) medium containing 1% sucrose, 0.8% agar supplemented with 100 mg/l myo-inositol, 1 mg/l thiamine hydrochloride, 0.5 mg/l pyridoxine hydrochloride and 0.5 mg/l nicotinic acid. For stratification, plates were kept in the dark at 4°C for 72 h and then placed under defined growth conditions. All plants were grown *in vitro* in plant growth chambers under a photoperiod of 16 h light (35 μmol m^−2^ s^−1^) at 20°C and 8 h dark at 18°C. After 10 days seedlings were transferred from agar plates into 200 ml glass culture vessels (5.5 cm diameter × 10 cm high, glass jars with magenta B caps) containing 8 ml sterile liquid medium. Plants grown in liquid cultures were incubated on rotary platform shakers at 120 rpm. After 17 days plants were harvested (28^th^ day of culture), leaves and roots were frozen separately in liquid nitrogen and stored at −80°C. All genotypes were grown in three biological replicates (in independent flasks). The growth conditions were monitored by a HOBO U12 data logger (Onset Computer Corporation, Bourne, MA) that recorded the parameters (temperature, light intensity and relative humidity) in an interval at every five minutes.

### Preparation of methanol extracts from Arabidopsis roots

The root tissue was homogenized using steel beads and sonication. The coumarins were extracted at 4°C with 80% methanol. After 24 h two sets of methanol extracts were centrifuged for 20 min at 13000 rpm, one set was additionally subjected to enzymatic hydrolysis using β-glucosidase from almonds (Sigma-Aldrich) dissolved in acetate buffer according to modified protocol of [56].

### Scopoletin and scopolin quantification by High-Performance Liquid Chromatography (HPLC)

The methanol extracts of Arabidopsis roots with and without enzymatic treatment were analyzed (Figure 2) using a Perkin Elmer series 200 HPLC system comprising of a quaternary LC pump, autosampler, column oven and a UV detector. All samples were filtered with 0.22 μm filters before loading. The volume injected was 10 μl. Gradient elution on Perkin Elmer C18 column SC18 (250×4.6 mm) was performed at flow rate of 0.7 ml/min with the following solvent system: (A) 50 mM ammonium acetate pH 4.5, (B) Methanol: starting from 30% B for 2 min, 30–80% B in 40 min followed by isocratic elution and column regeneration. The fluorescence detector was based on absorbance at 340 nm excitation wavelength and emission at 460 nm. The data analysis consisted of scopoletin and scopolin relative analysis (area percent of total chromatogram).

### Scopoletin identification by Gas Chromatography/Mass Spectrometry (GC/MS)

The HPLC fractions containing scopoletin peak were collected and scopoletin identification was confirmed (Figure 3A) with Gas Chromatography/Mass Spectrometry (GC/MS) by comparison to spectrum library (Figure 3B). GC/MS analysis was performed using a Perkin-Elmer GC XL Gas Chromatograph interfaced to a Mass Spectrometer equipped with an Elite-5MS (5% diphenyl/ 95% dimethyl polysiloxane) fused to a capillary column (30 × 0.25 μm ID × 0.25 μm df). For GC/MS detection, an electron ionization system operated in electron impact mode with an ionization energy of 70 eV. Helium gas was used as a carrier gas at a constant flow rate of 1 ml/min, and an injection volume of 2 μl was employed (a split ratio of 10:1). The ion-source temperature was 250°C, the oven temperature was programmed from 100°C (isothermal for 5 min), with an increase of 10°C/min to 300°C. Mass spectra were taken at 70 eV; a scan interval of 0.5 s and fragments from 30 to 450 Da. The solvent delay was 1 to 2 min, and the total GC/MS running time was 38 min. The mass-detector used in this analysis was Turbo-Mass Gold-Perkin-Elmer, and the MS software Turbo-Mass ver-5.1.

### Quantitative traits

Coumarins were quantified in the methanol root extracts of three biological replicates (cultivated in independent flasks) of all AI-RILs individuals. Methanol extracts subjected to enzymatic hydrolysis were used for scopoletin quantification, while scopolin contents were determined in methanol extracts without hydrolysis.

### Quantitative genetic analyses

The scopolin and scopoletin mean values for each AI-RILs were used in QTL mapping and trait correlation analysis. The regression equation and R^2^ were calculated by plotting scopolin and scopoletin mean values against one another in Scatterplot (Microsoft Excel). The broad sense heritability (*H*^*2*^) was estimated according to the formula *H*^*2*^ *= V*_*G*_*/(V*_*G*_ *+ V*_*E*_*)*, where *V*_*G*_ is the among-genotype variance component and *V*_*E*_ is the residual (error) variance.

### QTL analyses in the AI-RIL population

Statistical analysis of phenotypic data was performed by Shapiro-Wilk normality test. Phenotypic data is normally distributed at the significance level α = 0.05. QTL mapping was performed using R software (A Core Team, 2012, www.R-project.org) with R/qtl package [57,58]; http://www.rqtl.org/). QTL mapping was performed with Simple Interval Mapping (SIM) (data not shown) followed by the Multiple QTL mapping (MQM) procedure. The QTLs with the highest logarithm of odds (LOD) scores detected by SIM were subsequently used to make the QTL model by the MQM. The final QTL model was done with the backward elimination of cofactors with the window size 10 cM and maximum number of cofactors 5. Significance threshold (LOD) values (P <0.05) for the QTL presence was estimated from 10 000 permutations and is 3.4. “Addint” function has been used to add pairwise interaction, one at a time, to a multiple-QTL model. No interaction has been detected.

### Candidate genes selection

The physical positions of genes annotated to be involved in coumarin biosynthetic process (Plant Metabolic Network, http://plantcyc.org/) were checked according to TAIR (http://www.arabidopsis.org/). To reveal other candidate genes possibly underlying detected QTLs, a list of candidates was constructed using the following criteria: (1) genes encoding enzymes belonging to families involved in coumarins biosynthesis and genes encoding transcription factors that might be induced by environmental stresses (http://www.arabidopsis.org/); (2) genes that are expressed in roots (http://bar.utoronto.ca/). The list of potential candidates was compiled by searching TAIR (http://www.arabidopsis.org/) and Arabisopsis eFP Browser (http://bar.utoronto.ca/) (Table 4).

### Statistical analysis

All treatments included at least three (or two in case of parental lines used in the genetic mapping) biological replicates. Data processing and statistical analyses (one way ANOVA, post-hoc test: least significant difference test [LSD]) were carried out using Microsoft Excel. Error bars representing standard deviation (SD) are shown in the figures; the data presented are means.

### DNA samples preparation and sequencing

The RNeasy® Plant Mini Kit (Qiagen) was used following the instructions of the manufacturer and including on-column DNA digestion step with the RNase-Free DNase Set (Qiagen) to eliminate genomic DNA contamination. 0.5 μg of RNA was used for reverse transcription by Maxima First Strand cDNA Synthesis Kit (Thermo Scientific). The amplification of genes coding sequences was carried out in a 20 μl reaction mixture containing cDNA synthetized from RNA isolated from roots, 0.4 U of Platinum® *Taq* DNA Polymerase (Invitrogen), 200 μM dNTP, 1 μM primers, and 1 × PCR Buffer and 1.5 mM Mg^2+^. The reaction mixture was denatured at 94°C for 2 min, and then the PCR amplification was performed using 34 cycles of 94°C for 30 sec, 52°C for 30 sec, and 72°C for 90 sec in the Thermal Cycler C1000 Touch (Bio-Rad). Gene-specific primers used for AT5G53990 UDP-glycosyltransferase amplification were 5′- ATGGGCCAAAATTTTCACGCT -3′ and 5′- TCATTCAAGATTTGTATCGTTGACT-3′ and for AT3G28740 CYP81D11 5′- ATGTCATCAACAAAGACAATAATGG-3′ and 5′- TTATGGACAAGAAGCATCTAAAACC-3′. PCR products were cloned into pCR8 vector (Invitrogen). For plasmid amplification and maintenance, the *Escherichia coli* strain One Shot® (Invitrogen) was used. Positive clones were sequenced using vector specific primers M13fwd and M13rev and BigDye® Terminator v3.1 (Life Technologies). Sequencing reaction products were separated and analyzed by 3730xl DNA Analyzer. All sequences were aligned using CLUSTALW [59].

### Availability of supporting data

The data sets supporting the results of this article are included within the article and its additional files.

## Additional files

Additional file 1: Figure S1. The position of known loci involved in scopolin and scopoletin biosynthesis. Additional file 2: Figure S2. Multiple Sequence Alignment of coding sequences of *AtCYP81D11* gene produced by CLUSTALW. Additional file 3: Figure S3. Multiple Sequence Alignment of coding sequences of *AtUDP-glycosyltransferase* gene produced by CLUSTALW.

## Abbreviations

2OGD: 2-oxoglutarate-dependent dioxygenase
4CL1: 4-coumarate:CoA ligase 1
4CL2: 4-coumarate:CoA ligase 2
4CL3: 4-coumarate:CoA ligase 3
4CL5: 4-coumarate:CoA ligase 5
ACOS5: Acyl-CoA synthetase 5
AI-RILs: Advanced intercross recombinant inbred lines
C3H: p-coumaroyl 3′-hydroxylase
CCoAOMT1: Caffeoyl coenzyme A dependent O-methyltransferase 1
CCoAOMT7: Caffeoyl coenzyme A dependent O-methyltransferase 7
CYP: Cytochrome P450 superfamily of monooxygenases
F6′H1: Feruloyl-CoA 6′-hydroxylase 1
F6′H2: Feruloyl-CoA 6′-hydroxylase 2
GC/MS: Gas Chromatography/Mass Spectrometry
HCT: Shikimate O-hydroxycinnamoyltransferase
HPLC: High-performance liquid chromatography
LOD: Logarithm of odds
MS: Murashige and Skoog medium
MQM: Multiple QTL mapping
MYB: Superfamily of transcription factors
NASC: Nottingham Arabidopsis stock centre
OMT1: Caffeate O-methyltransferase 1
TSM1: Tapetum-specific O- methyltransferase
PVE: Phenotypic variance explained
SIM: Simple interval mapping
QTL: Quantitative trait loci
WRKY: Superfamily of transcription factors
