# Supplementary material for: Identification of QTLs affecting scopolin and scopoletin biosynthesis in Arabidopsis thaliana
Source: BMC Plant Biol. 2014 Oct 18;14:280. doi: 10.1186/s12870-014-0280-9 (PMC4252993; doi:10.1186/s12870-014-0280-9)
Supplement: Additional file 1: Figure S1. — The position of known loci involved in scopolin and scopoletin biosynthesis. [file 12870_2014_280_MOESM1_ESM.pdf]

Additional file 1:

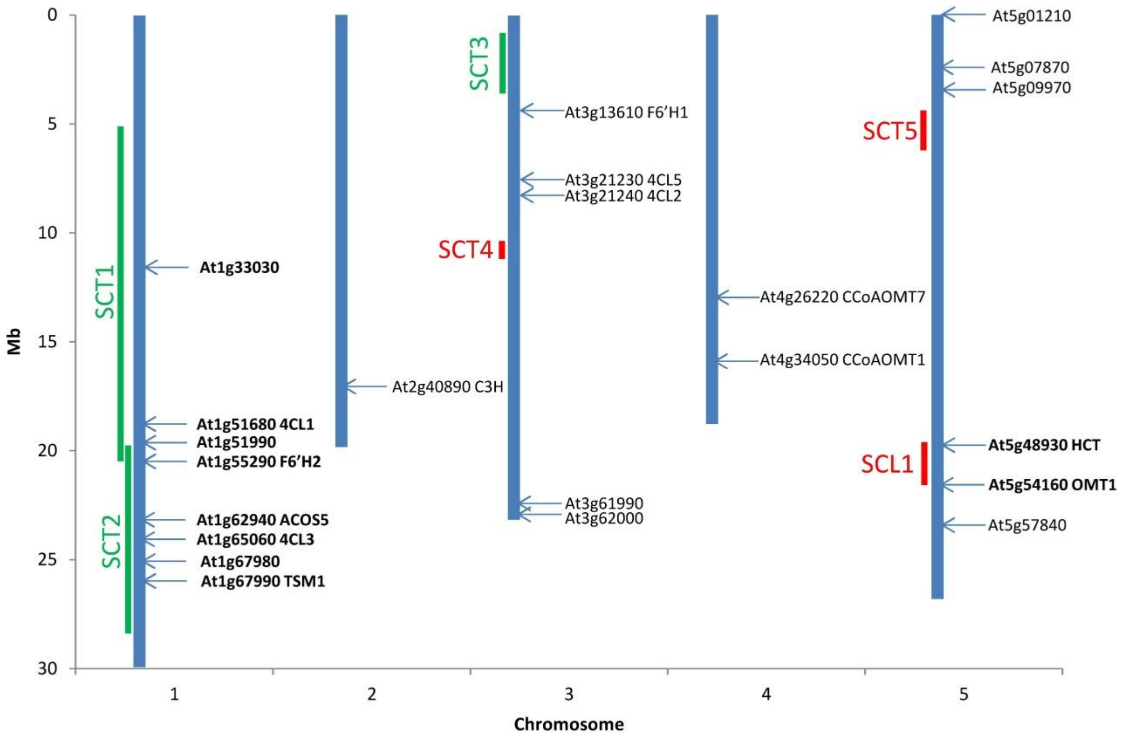

Figure S1. The position of known loci involved in scopolin and scopoletin biosynthesis. Mapped QTLs are highlighted in green and red colours. QTLs characterized by the highest LOD score values are highlighted in red. Loci co-localizing with QTLs are highlighted in bold.
