# Supplementary material for: Identification of QTLs affecting scopolin and scopoletin biosynthesis in Arabidopsis thaliana
Source: BMC Plant Biol. 2014 Oct 18;14:280. doi: 10.1186/s12870-014-0280-9 (PMC4252993; doi:10.1186/s12870-014-0280-9)
Supplement: Additional file 2: Figure S2. — Multiple Sequence Alignment of coding sequences of AtCYP81D11 gene produced by CLUSTALW. [file 12870_2014_280_MOESM2_ESM.pdf]

Additional file 2:

```
Kond            ATGTCATCAACAAAGACAATAATGGAAACTATATACCTAATTCTCTCTCTCTTCTTCTTC 60
Ler             ATGTCATCAACAAAGACAATAATGGAAACTATATACCTAATTCTCTCTCTCTTCTTCTTC 60
Est             ATGTCATCAACAAAGACAATAATGGAAACTATATACCTAATTCTCTCTCTCTTCTTCTT- 59
Col-0           ATGTCATCAACAAAGACAATAATGGAAACTATATACCTAATTCTCTCTCTCTTCTTCTTC 60
Est-1           ATGTCATCAACAAAGACAATAATGGAAACTATATACCTAATTCTCTCTCTCTTCTTCTTC 60
Tsu-1           ATGTCATCAACAAAGACAATAATGGAAACTATATACCTAATTCTCTCTCTCTTCTTCTTC 60
An-1            ATGTCATCAACAAAGACAATAATGGAAACTATATACCTAATTCTCTCTCTCTTCTTCTTC 60
Est-1*          ATGTCATCAACAAAGACAATAATGGAAACTATATACCTAATTCTCTCTCTCTTCTTCTTC 60
                *********************************************************** 

Kond            TTCATCTTCCTATCTCTCAAACTCGTGTTCGGCCCACGGCCACGTAAACTAAACCTACCG 120
Ler             TTCATCTTCCTATCTCTCAAACTCGTGTTCGGCCCACGGCCACGTAAACTAAACCTACCG 120
Est             --CATCTTCCTATCTCTCAAACTCTTGTTCGGCCCACGGCCGCGTAAACTAAACCTACCG 117
Col-0           TTCATCTTCCTATCTCTCAAACTCTTGTTCGGACCACGGCCGCGTAAACTAAACCTACCG 120
Est-1           TTCATCTTCCTATCTCTCAAACTCTTGTTCGGACCACGGCCGCGTAAACTAAACCTACCG 120
Tsu-1           TTCATCTTCCTATCTCTCAAACTCTTGTTCGGACCACGGCCGCGTAAACTAAACCTACCG 120
An-1            TTCATCTTCCTATCTCTCAAACTCTTGTTCGGACCACGGCCGCGTAAACTAAACCTACCG 120
Est-1*          TTCATCTTCCTATCTCTCAAACTCTTGTTCGGACCACGGCCGCGTAAACTAAACCTACCG 120
                  ********************** ******* ******** ******************

Kond            CCGAGTCCGAGTCGACCATTTCCGATCATCGGACACCTCCACCTTCTAAAGCTACCACTC 180
Ler             CCGAGTCCGAGTCGACCATTTCCGATCATCGGACACCTCCACCTTCTAAAGCTACCACTC 180
Est             CCTAGTCCGAGTCGACCATTTCCGGTCATCGGACACCTCCACCTTCTAAAGCTACCACTC 177
Col-0           CCGAGTCCGAGTCGACCATTTCCGATCATCGGACACCTCCACCTTCTAAAGCTACCACTC 180
Est-1           CCGAGTCCGAGTCGACCATTTCCGATCATCGGACACCTCCACCTTCTAAAGCTACCACTC 180
Tsu-1           CCGAGTCCGAGTCGACCATTTCCGATCATCGGACACCTCCACCTTCTAAAGCTACCACTC 180
An-1            CCGAGTCCGAGTCGACCATTTCCGATCATCGGACACCTCCACCTTCTAAAGCTACCACTC 180
Est-1*          CCGAGTCCGAGTCGACCATTTCCGATCATCGGACACCTCCACCTTCTAAAGCTACCACTC 180
                ** ********************* ***********************************
```

```
Kond      CACCGTAGATTTCTCTCTCTCTCAGAATCTTTAAACAACGCCAAGATATTCTCTCTTCGC 240
Ler       CACCGTAGATTTCTCTCTCTCTCAGAATCTTTAAACAACGCCAAGATATTCTCTCTTCGC 240
Est       CACCGTAGATTTCTCTCTCTCTCAGAATCTTTAAACGACGCCAAAATATTCTCTCTTCGC 237
Col-0     CACCGTAGATTTCTCTCTCTCTCAGAATCTTTAAACAACGCCAAGATTTTCTCTCTCAGC 240
Est-1     CACCGTAGATTTCTCTCTCTCTCAGAATCTTTAAACAACGCCAAGATTTTCTCTCTCAGC 240
Tsu-1     CACCGTAGATTTCTCTCTCTCTCAGAATCTTTAAACAACGCCAAGATTTTCTCTCTCAGC 240
An-1      CACCGTAGATTTCTCTCTCTCTCAGAATCTTTAAACAACGCCAAGATTTTCTCTCTCAGC 240
Est-1*    CACCGTAGATTTCTCTCTCTCTCAGAATCTTTAAACAACGCCAAGATTTTCTCTCTCAGC 240
          ************************************ ******* ** ********  **

Kond      CTCGGCTCTCGACTAGTTTTTGTTGTTTCTTCACACGCCGTGGCGGAAGAATGTTTCACT 300
Ler       CTCGGCTCTCGACTAGTTTTTGTTGTTTCTTCACACGCCGTGGCGGAAGAATGTTTCACT 300
Est       CTCGGCTCTCGTCTAGTTTTTGTTGTTTCTTCTCACGCCGTCGCTGAAGAATGTTTCACT 297
Col-0     CTCGGCTCTCGTCTAGTTTTTGTTGTTTCTTCTCACGCCGTCGCGGAAGAATGTTTCACT 300
Est-1     CTCGGCTCTCGTCTAGTTTTTGTTGTTTCTTCTCACGCCGTCGCGGAAGAATGTTTCACT 300
Tsu-1     CTCGGCTCTCGTCTAGTTTTTGTTGTTTCTTCTCACGCCGTCGCGGAAGAATGTTTCACT 300
An-1      CTCGGCTCTCGTCTAGTTTTTGTTGTTTCTTCTCACGCCGTCGCGGAAGAATGTTTCACT 300
Est-1*    CTCGGCTCTCGTCTAGTTTTTGTTGTTTCTTCTCACGCCGTCGCGGAAGAATGTTTCACT 300
          *********** ******************** ******** ** ***************

Kond      AAGAACGACGTCGTGTTAGCTAACCGGCCGGAGTTTCTCGTCGGGAAACATATAGGGTAC 360
Ler       AAGAACGACGTCGTGTTAGCTAACCGGCCGGAGTTTCTCGTCGGGAAACATATAGGGTAC 360
Est       AAGAACGACGTCGTGTTAGCTAACCGGCCGGAGTTTCTCGTCGGGAAACATATAGGGTAC 357
Col-0     AAGAACGACGTCGTGTTAGCTAACCGGCCGGAGTTTCTCGTCGGGAAACATATAGGGTAC 360
Est-1     AAGAACGACGTCGTGTTAGCTAACCGGCCGGAGTTTCTCGTCGGGAAACATATAGGGTAC 360
Tsu-1     AAGAACGACGTCGTGTTAGCTAACCGGCCGGAGTTTCTCGTCGGGAAACATATAGGGTAC 360
An-1      AAGAACGACGTCGTGTTAGCTAACCGGCCGGAGTTTCTCGTCGGGAAACATATAGGGTAC 360
Est-1*    AAGAACGACGTCGTGTTAGCTAACCGGCCGGAGTTTCTCGTCGGGAAACATATAGGGTAC 360
          ************************************************************
```

```
Kond      AACTCTACGACCATGGTTGGCGCGGCTTAAGGAGATAGTTGGAGGAATCTTCGACGTATT 420
Ler       AACTCTACGACCATGGTTGGCGCGGCTTAAGGAGATAGTTGGAGGAATCTTCGACGTATT 420
Est       AACTCTACGACCATGGTTGGCGCGGCTTAAGGAGATAGTTGGAGGAATCTTCGACGTATT 417
Col-0     AACTCTACGACCATGGTTGGTGCGGCTTATGGAGATAGTTGGAGGAATCTTCGACGTATT 420
Est-1     AACTCTACGACCATGGTTGGTGCGGCTTATGGAGATAGTTGGAGGAATCTTCGACGTATT 420
Tsu-1     AACTCTACGACCATGGTTGGTGCGGCTTATGGAGATAGTTGGAGGAATCTTCGACGTATT 420
An-1      AACTCTACGACCATGGTTGGTGCGGCTTATGGAGATAGTTGGAGGAATCTTCGACGTATT 420
Est-1*    AACTCTACGACCATGGTTGGTGCGGCTTATGGAGATAGTTGGAGGAATCTTCGACGTATT 420
          ******************** ******** ******************************

Kond      GGCACCATTGAGATCTTCTCGTCTCTTAGGCTTAATAGCTTCGTATCTATCCGTCACGAT 480
Ler       GGCACCATTGAGATCTTCTCGTCTCTTAGGCTTAATAGCTTCGTATCTATCCGTCACGAT 480
Est       GGCACCATTGAGATCTTCTCGTCTCTTAGGCTTAATAGCTTCGTATCTATCCGTCACGAT 477
Col-0     GGCACCATTGAGATCTTCTCGTCTCTTAGGCTTAATAGCTTCGTATCTATCCGTCAAGAT 480
Est-1     GGCACCATTGAGATCTTCTCGTCTCTTAGGCTTAATAGCTTCGTATCTATCCGTCAAGAT 480
Tsu-1     GGCACCATTGAGATCTTCTCGTCTCTTAGGCTTAATAGCTTCGTATCTATCCGTCAAGAT 480
An-1      GGCACCATTGAGATCTTCTCGTCTCTTAGGCTTAATAGCTTCGTATCTATCCGTCAAGAT 480
Est-1*    GGCACCATTGAGATCTTCTCGTCTCTTAGGCTTAATAGCTTCGTATCTATCCGTCAAGAT 480
          ******************************************************** ***

Kond      GAGATCCGACGGCTCATAATTTGTCTAGCGAAAAACTCTCAACATGGGTTTGTGAAAGTG 540
Ler       GAGATCCGACGGCTCATAATTTGTCTAGCGAAAAACTCTCAACATGGGTTTGTGAAAGTG 540
Est       GAGATCCGACGGCTCATAATTTGTCTAGCGAAAAACTCTCAACATGGGTTTGTGAAAGTG 537
Col-0     GAGATCCGACGGCTCATAATTTGTCTAGCGAAAAACTCTCAACATGGGTTTGTGAAAGTG 540
Est-1     GAGATCCGACGGCTCATAATTTGTCTAGCGAAAAACTCTCAACATGGGTTTGTGAAAGTG 540
Tsu-1     GAGATCCGACGGCTCATAATTTGTCTAGCGAAAAACTCTCAACATGGGTTTGTGAAAGTG 540
An-1      GAGATCCGACGGCTCATAATTTGTCTAGCGAAAAACTCTCAACATGGGTTTGTGAAAGTG 540
Est-1*    GAGATCCGACGGCTCATAATTTGTCTAGCGAAAAACTCTCAACAAGAGTTTGTGAAAGTG 540
          ******************************************** * *************
```

```
Kond            GAGATGAAACCATTGTTTATGTGTTTAACTATCAACAACATCATTAGAATGGTGGCCGGA 600
Ler             GAGATGAAACCATTGTTTATGTGTTTAACTATCAACAACATCATTAGAATGGTGGCCGGA 600
Est             GAGATGAAACCATTGTTTATGTGTTTAACTATCAACAACATCATTAGAATGGTGGCCGGA 597
Col-0           GAGATGAAACCATTGTTTATGTGTTTAACTATCAACAACATCATTAGAATGGTGGCCGGA 600
Est-1           GAGATGAAACCATTGTTTATGTGTTTAACTATCAACAACATCATTAGAATGGTGGCCGGA 600
Tsu-1           GAGATGAAACCATTGTTTATGTGTTTAACTATCAACAACATCATTAGAATGGTGGCCGGA 600
An-1            GAGATGAAACCATTGTTTATGTGTTTAACTATCAACAACATCATTAGAATGGTGGCCGGA 600
Est-1*          GAGATGAAACCATTGTTTATGTGTTTAACTATCAACAACATCATTAGAATGGTGGCCGGA 600
                ************************************************************

Kond            AAAAGATTCTACGGTGATGGAACAGAGAACGACAACGAGGCTAAACACGTGAGACAGCTA 660
Ler             AAAAGATTCTACGGTGATGGAACAGAGAACGACAACGAGGCTAAACACGTGAGACAGCTA 660
Est             AAAAGATTCTACGGTGATGGAACAGAGAACGACAACGAGGCTAAACACGTGAGACAGCTA 657
Col-0           AAAAGATTCTACGGTGATGGAACAGAGAACGACAACGAGGCTAAACACGTGAGACAGCTA 660
Est-1           AAAAGATTCTACGGTGATGGAACAGAGAACGACAACGAGGCTAAACACGTGAGACAGCTA 660
Tsu-1           AAAAGATTCTACGGTGATGGAACAGAGAACGACAACGAGGCTAAACACGTGAGACAGCTA 660
An-1            AAAAGATTCTACGGTGATGGAACAGAGAACGACAACGAGGCTAAACACGTGAGACAGCTA 660
Est-1*          AAAAGATTCTACGGTGATGGAACAGAGAACGACAACGAGGCAAAACACGTGAGACAGTTG 660
                ***************************************** *************** * 

Kond            ATTGCCGAGGTAGTTGTTAGCGGCGGCGCAGGGAACGCAGCAGACTACTTCCCTATCCTT 720
Ler             ATTGCCGAGGTAGTTGTTAGCGGCGGCGCAGGGAACGCAGCAGACTACTTCCCTATCCTT 720
Est             ATTGCCGAGGTAGTTGTTAGCGGCGGCGCAGGGAACGCAGCAGACTACTTCCCTATCCTT 717
Col-0           ATTGCCGAGGTAGTTGTTAGCGGCGGCGCAGGGAACGCAGCAGACTACTTCCCTATCCTT 720
Est-1           ATTGCCGAGGTAGTTGTTAGCGGCGGCGCAGGGAACGCAGCAGACTACTTCCCTATCCTT 720
Tsu-1           ATTGCCGAGGTAGTTGTTAGCGGCGGCGCAGGGAACGCAGCAGACTACTTCCCTATCCTT 720
An-1            ATTGCCGAGGTAGTTGTTAGCGGCGGCGCAGGGAACGCAGCAGACTACTTCCCTATCCTT 720
Est-1*          ATCGCCGAGGTAGTTGTTAGCGGCGGCGCGGGGAACGCAGCAGACTACTTCCCTATCCTT 720
                ** ************************** ******************************
```

```
Kond      CGTTACGTAACGAACTATGAGAAACATGTTAAGAAGTTAGCAGGTCGTGTTGATGAGTTT 780
Ler       CGTTACGTAACGAACTATGAGAAACATGTTAAGAAGTTAGCAGGTCGTGTTGATGAGTTT 780
Est       CGTTACGTAACGAACTATGAGAAACATGTTAAGAAGTTAGCAGGTCGTGTTGATGAGTTT 777
Col-0     CGTTACGTAACGAACTATGAGAAACATGTTAAGAAGTTAGCAGGTCGTGTTGATGAGTTT 780
Est-1     CGTTACGTAACGAACTATGAGAAACATGTTAAGAAGTTAGCAGGTCGTGTTGATGAGTTT 780
Tsu-1     CGTTACGTAACGAACTATGAGAAACATGTTAAGAAGTTAGCAGGTCGTGTTGATGAGTTT 780
An-1      CGTTACGTAACGAACTATGAGAAACATGTTAAGAAGTTAGCAGGTCGTGTTGATGAGTTT 780
Est-1*    CGTTATGTAACGAACTATGAGAAACATGTTAAGAAGTTAGCAGGTCGTGTTGATGAGTTT 780
          ***** ******************************************************

Kond      TTGCAAAGTCTAGTTAACGAGAAACGTGTGGAGAAAGTAAAAGGTAACACAATGATCGAT 840
Ler       TTGCAAAGTCTAGTTAACGAGAAACGTGTGGAGAAAGTAAAAGGTAACACAATGATCGAT 840
Est       TTGCAAAGTCTAGTTAACGAGAAACGTGTGGAGAAAGTAAAAGGTAACACAATGATCGAT 837
Col-0     TTGCAAAGTCTAGTTAACGAGAAACGTGTGGAGAAAGTAAAAGGTAACACAATGATCGAT 840
Est-1     TTGCAAAGTCTAGTTAACGAGAAACGTGTGGAGAAAGTAAAAGGTAACACAATGATCGAT 840
Tsu-1     TTGCAAAGTCTAGTTAACGAGAAACGTGTGGAGAAAGTAAAAGGTAACACAATGATCGAT 840
An-1      TTGCAAAGTCTAGTTAACGAGAAACGTGTGGAGAAAGTAAAAGGTAACACAATGATCGAT 840
Est-1*    TTGCAAAGTCTAGTGAACGAGAAACGTGAGGAGAAAGTAAAAGGTAACACAATGATCGAT 840
          ************** ************* *******************************

Kond      CATTTACTTTCTCTACAAGAAACTCAGCCTGATTACTACACGGATGTGATCATCAAAGGA 900
Ler       CATTTACTTTCTCTACAAGAAACTCAGCCTGATTACTACACGGATGTGATCATCAAAGGA 900
Est       CATTTACTTTCTCTACAAGAAACTCAGCCTGATTACTACACGGATGTGATCATCAAAGGA 897
Col-0     CATTTACTTTCTCTACAAGAAACTCAGCCTGATTACTACACGGATGTGATCATCAAAGGA 900
Est-1     CATTTACTTTCTCTACAAGAAACTCAGCCTGATTACTACACGGATGTGATCATCAAAGGA 900
Tsu-1     CATTTACTTTCTCTACAAGAAACTCAGCCTGATTACTACACGGATGTGATCATCAAAGGA 900
An-1      CATTTACTTTCTCTACAAGAAACTCAGCCTGATTACTACACGGATGTGATCATCAAAGGA 900
Est-1*    CATTTACTTTCTCTACAAGAAACTCAGCCTGATTACTACACTGATGTGATCATCAAAGGA 900
          ***************************************** ******************
```

```
Kond          ATCATACTTGTTATGATACTTGCCGGGACTGATACATCAGCTGGAACGTTAGAATGGGCG 960
Ler           ATCATACTTGTTATGATACTTGCCGGGACTGATACATCAGCTGGAACGTTAGAATGGGCG 960
Est           ATCATACTTGTTATGATACTTGCCGGGACTGATACATCAGCTGGAACGTTAGAATGGGCG 957
Col-0         ATCATACTTGTTATGATACTTGCCGGGACTGATACATCAGCTGGAACGTTAGAATGGGCG 960
Est-1         ATCATACTTGTTATGATACTTGCCGGGACTGATACATCAGCTGGAACGTTAGAATGGGCG 960
Tsu-1         ATCATACTTGTTATGATACTTGCCGGGACTGATACATCAGCTGGAACGTTAGAATGGGCG 960
An-1          ATCATACTTGTTATGATACTTGCCGGGACTGATACATCAGCTGGAACGTTAGAATGGGCG 960
Est-1*        ATCATACTTGTTATGATACTTGCCGGGACTGATACATCAGCTGGAACGTTAGAATGGGCG 960
              ************************************************************

Kond          ATGTCGAATTTGTTGAACCATCCAGAAGTATTACGAAAGGCAAAGACCGAAATCGACGAC 1020
Ler           ATGTCGAATTTGTTGAACCATCCAGAAGTATTACGAAAGGCAAAGACCGAAATCGACGAC 1020
Est           ATGTCGAATTTGTTGAACCATCCAGAAGTATTACGAAAGGCAAAGACCGAAATCGACGAC 1017
Col-0         ATGTCGAATTTGTTGAACCATCCAGAAGTATTACGAAAGGCAAAGACCGAAATCGACGAC 1020
Est-1         ATGTCGAATTTGTTGAACCATCCAGAAGTATTACGAAAGGCAAAGACCGAAATCGACGAC 1020
Tsu-1         ATGTCGAATTTGTTGAACCATCCAGAAGTATTACGAAAGGCAAAGACCGAAATCGACGAC 1020
An-1          ATGTCGAATTTGTTGAACCATCCAGAAGTATTACGAAAGGCAAAGACCGAAATCGACGAC 1020
Est-1*        ATGTCGAATTTGTTGAACCATCCAGAAGTATTACGAAAGGCAAAGACCGAAATCGACGAC 1020
              ************************************************************

Kond          CAAATCGGTGTAGACCGGTTAGTAGAGGAACAAGACATTGTGAAACTTCCTTACCTTCAG 1080
Ler           CAAATCGGTGTAGACCGGTTAGTAGAGGAACAAGACATTGTGAAACTTCCTTACCTTCAG 1080
Est           CAAATCGGTGTAGACCGGTTAGTAGAGGAACAAGACATTGTGAAACTTCCTTACCTTCAG 1077
Col-0         CAAATCGGTGTAGACCGGTTAGTAGAGGAACAAGACATTGTGAAACTTCCTTACCTTCAG 1080
Est-1         CAAATCGGTGTAGACCGGTTAGTAGAGGAACAAGACATTGTGAAACTTCCTTACCTTCAG 1080
Tsu-1         CAAATCGGTGTAGACCGGTTAGTAGAGGAACAAGACATTGTGAAACTTCCTTACCTTCAG 1080
An-1          CAAATCGGTGTAGACCGGTTAGTAGAGGAACAAGACATTGTGAAACTTCCTTACCTTCAG 1080
Est-1*        CAAATCGGTGTAGACCGGTTAATAGAGGAACAAGACATTGTGAAACTTCCTTACCTTCAG 1080
              ********************* **************************************
```

```
Kond        CACATTGTGTCAGAGACTTTACGGCTTTATCCGGTGGCTCCAATGCTTTTACCCCACTTA 1140
Ler         CACATTGTGTCAGAGACTTTACGGCTTTATCCGGTGGCTCCAATGCTTTTACCCCACTTA 1140
Est         CACATTGTGTCAGAGACTTTACGGCTTTATCCGGTGGCTCCAATGCTTTTACCCCACTTA 1137
Col-0       CACATTGTGTCAGAGACTTTACGGCTTTATCCGGTGGCTCCAATGCTTTTACCCCACTTA 1140
Est-1       CACATTGTGTCAGAGACTTTACGGCTTTATCCGGTGGCTCCAATGCTTTTACCCCACTTA 1140
Tsu-1       CACATTGTGTCAGAGACTTTACGGCTTTATCCGGTGGCTCCAATGCTTTTACCCCACTTA 1140
An-1        CACATTGTGTCAGAGACTTTACGGCTTTATCCGGTGGCTCCAATGCTTTTACCCCACTTA 1140
Est-1*      CACATTGTGTCAGAGACTTTACGGCTTTATCCAGTGGCTCCAATGCTTTTACCCCACTTA 1140
            ******************************** ***************************

Kond        GCATCAGAAGATTGTATAGTTGATGGCTATGACGTTCCACGCGGCACAATCATCTTGGTG 1200
Ler         GCATCAGAAGATTGTATAGTTGATGGCTATGACGTTCCACGCGGCACAATCATCTTGGTG 1200
Est         GCATCAGAAGATTGTATAGTTGATGGCTATGACGTTCCACGCGGCACAATCATCTTGGTG 1197
Col-0       GCATCAGAAGATTGTATAGTTGATGGCTATGACGTTCCACGCGGCACAATCATCTTGGTG 1200
Est-1       GCATCAGAAGATTGTATAGTTGATGGCTATGACGTTCCACGCGGCACAATCATCTTGGTG 1200
Tsu-1       GCATCAGAAGATTGTATAGTTGATGGCTATGACGTTCCACGCGGCACAATCATCTTGGTG 1200
An-1        GCATCAGAAGATTGTATAGTTGATGGCTATGACGTTCCACGCGGCACAATCATCTTGGTG 1200
Est-1*      GCATCAGAAAATTGTGTAGTTGATGGCTATGACGTTCCACAAGGCACAATCATCTTGGTG 1200
            ********* ***** ************************  ******************

Kond        AATGCATGGGCCATCCATAGAGATCCAAAGTTATGGGAAGAGCCAGAAAAATTTAAGCCG 1260
Ler         AATGCATGGGCCATCCATAGAGATCCAAAGTTATGGGAAGAGCCAGAAAAATTTAAGCCG 1260
Est         AATGCATGGGCCATCCATAGAGATCCAAAGTTATGGGAAGAGCCAGAAAAATTTAAGCCG 1257
Col-0       AATGCATGGGCCATCCATAGAGATCCAAAGTTATGGGAAGAGCCAGAAAAATTTAAGCCG 1260
Est-1       AATGCATGGGCCATCCATAGAGATCCAAAGTTATGGGAAGAGCCAGAAAAATTTAAGCCG 1260
Tsu-1       AATGCATGGGCCATCCATAGAGATCCAAAGTTATGGGAAGAGCCAGAAAAATTTAAGCCG 1260
An-1        AATGCATGGGCCATCCATAGAGATCCAAAGTTATGGGAAGAGCCAGAAAAATTTAAGCCG 1260
Est-1*      AATGCATGGGCCATTCATAGAGATCCAAAGTTATGGGAAGAGCCAGAAAAGTTTAAGCCG 1260
            ************** *********************************** *********
```

```
Kond        GAGAGGTTTGAGAAAAAAGGAGAGGATAAAAAGTTGATGCCATTTGGGATTGGACGACGA 1320
Ler         GAGAGGTTTGAGAAAAAAGGAGAGGATAAAAAGTTGATGCCATTTGGGATTGGACGACGA 1320
Est         GAGAGGTTTGAGAAAAAAGGAGAGGATAAAAAGTTGATGCCATTTGGGATTGGACGACGA 1317
Col-0       GAGAGGTTTGAGAAAAAAGGAGAGGATAAAAAGTTGATGCCATTTGGGATTGGACGACGA 1320
Est-1       GAGAGGTTTGAGAAAAAAGGAGAGGATAAAAAGTTGATGCCATTTGGGATTGGACGACGA 1320
Tsu-1       GAGAGGTTTGAGAAAAAAGGAGAGGATAAAAAGTTGATGCCATTTGGGATTGGACGACGA 1320
An-1        GAGAGGTTTGAGAAAAAAGGAGAGGATAAAAAGTTGATGCCATTTGGGATTGGACGACGA 1320
Est-1*      GAGAGGTTTGAGAAAAAAGGAGAGGATAAAAAGTTGATGCCTTTTGGGATTGGACGACGA 1320
            ***************************************** ******************

Kond        TCTTGCCCTGGTTCAGGGCTAGCTCAACGGCTAGTGACTTTGGCTCTTGGATCATTGGTC 1380
Ler         TCTTGCCCTGGTTCAGGGCTAGCTCAACGGCTAGTGACTTTGGCTCTTGGATCATTGGTC 1380
Est         TCTTGCCCTGGTTCAGGGCTAGCTCAACGGCTAGTGACTTTGGCTCTTGGATCATTGGTC 1377
Col-0       TCTTGCCCTGGTTCAGGGCTAGCTCAACGGCTAGTGACTTTGGCTCTTGGATCATTGGTC 1380
Est-1       TCTTGCCCTGGTTCAGGGCTAGCTCAACGGCTAGTGACTTTGGCTCTTGGATCATTGGTC 1380
Tsu-1       TCTTGCCCTGGTTCAGGGCTAGCTCAACGGCTAGTGACTTTGGCTCTTGGATCATTGGTC 1380
An-1        TCTTGCCCTGGTTCAGGGCTAGCTCAACGGCTAGTGACTTTGGCTCTTGGATCATTGGTC 1380
Est-1*      TCTTGTCCTGGTTCAGGGCTAGCTCAACGGCTAGTGACCTTGGCTCTAGGATCGTTGGTA 1380
            ***** ******************************** ******** ***** *****

Kond        CAATGTTTTGAGTGGGAGAGGGTTGAAGAAAAGTATCTAGACATGAGAGAAAGTGAAAAA 1440
Ler         CAATGTTTTGAGTGGGAGAGGGTTGAAGAAAAGTATCTAGACATGAGAGAAAGTGAAAAA 1440
Est         CAATGTTTTGAGTGGGAGAGGGTTGAAGAAAAGTATCTAGACATGAGAGAAAGTGAAAAA 1437
Col-0       CAATGTTTTGAGTGGGAGAGGGTTGAAGAAAAGTATCTAGACATGAGAGAAAGTGAAAAA 1440
Est-1       CAATGTTTTGAGTGGGAGAGGGTTGAAGAAAAGTATCTAGACATGAGAGAAAGTGAAAAA 1440
Tsu-1       CAATGTTTTGAGTGGGAGAGGGTTGAAGAAAAGTATCTAGACATGAGAGAAAGTGAAAAA 1440
An-1        CAATGTTTTGAGTGGGAGAGGGTTGAAGAAAAGTATCTAGACATGAGAGAAAGTGAAAAA 1440
Est-1*      CAATGTTTTGAGTGGGAGAGAGTTGGAGAAAAGTATCTAGACATGAGAGAAAGTGAAAAA 1440
            ******************** **** **********************************
```

```
Kond            GGAACCACAATGCGAAAAGCTACATCGTTGCAAGCTATGTGTAAAGCTCGTCCCATTGTC 1500
Ler             GGAACCACAATGCGAAAAGCTACATCGTTGCAAGCTATGTGTAAAGCTCGTCCCATTGTC 1500
Est             GGAACCACAATGCGAAAAGCTACATCGTTGCAAGCTATGTGTAAAGCTCGTCCCATTGTC 1497
Col-0           GGAACCACAATGCGAAAAGCTACATCGTTGCAAGCTATGTGTAAAGCTCGTCCCATTGTC 1500
Est-1           GGAACCACAATGCGAAAAGCTACATCGTTGCAAGCTATGTGTAAAGCTCGTCCCATTGTC 1500
Tsu-1           GGAACCACAATGCGAAAAGCTACATCGTTGCAAGCTATGTGTAAAGCTCGTCCCATTGTC 1500
An-1            GGAACCACAATGCGAAAAGCTACATCGTTGCAAGCTATGTGTAAAGCTCGTCCCATTGTC 1500
Est-1*          GGAACCACGATGCGCAAAGCTACATCGTTGCAAGCTATGTGTAAAGCTCGTCCCATTGTC 1500
                ******** ***** *********************************************

Kond            CATAAGGTTTTAGATGCTTCTTGTCCATAA 1530
Ler             CATAAGGTTTTAGATGCTTCTTGTCCATAA 1530
Est             CATAAGGTTTTAGATGCTTCTTGTCCATAA 1527
Col-0           CATAAGGTTTTAGATGCTTCTTGTCCATAA 1530
Est-1           CATAAGGTTTTAGATGCTTCTTGTCCATAA 1530
Tsu-1           CATAAGGTTTTAGATGCTTCTTGTCCATAA 1530
An-1            CATAAGGTTTTAGATGCTTCTTGTCCATAA 1530
Est-1*          CATAAGGTTTTAGATGCTTCTTGTCCATAA 1530
                ******************************
```

Figure S2. Multiple Sequence Alignment of coding sequences of *AtCYP81D11* gene produced by CLUSTALW. The following sequences were taken from the 1001 Genomes Project database (www.1001genomes.org): Kond, L*er*, Est, Col-0, Est-1, Tsu-1, An-1 (Kas-2 sequence was not available in the database). The selected sequences originate from accessions, which were used in the initial screening of natural variation in scopolin and scopoletin accumulation. The Est-1* sequence was obtained after re-sequencing of Est-1 accession from our laboratory.
