# Supplementary material for: Identification of QTLs affecting scopolin and scopoletin biosynthesis in Arabidopsis thaliana
Source: BMC Plant Biol. 2014 Oct 18;14:280. doi: 10.1186/s12870-014-0280-9 (PMC4252993; doi:10.1186/s12870-014-0280-9)
Supplement: Additional file 3: Figure S3. — Multiple Sequence Alignment of coding sequences of AtUDP-glycosyltransferase gene produced by CLUSTALW. [file 12870_2014_280_MOESM3_ESM.pdf]

Additional file 3:

```
Col-0           ATGGGCCAAAATTTTCACGCTTTTATGTTCCCATGGTTCGCTTTTGGTCATATGACTCCA 60
Est             ATGGGCCAAAATTTTCACGCTTTTATGTTCCCATGGTTCGCTTTTGGTCATATGACTCCA 60
Kond            ATGGGCCAAAATTTTCACGCTTTTATGTTCCCATGGTTCGCTTTTGGTCATATGACTCCA 60
Tsu-1           ATGGGCCAAAATTTTCACGCTTTTATGTTCCCATGGTTCGCTTTTGGTCATATGACTCCA 60
An-1            ATGGGCCAAACGTTTCACGCTTTTATGTTCCCATGGTTTGCTTTTGGTCATATGACTCCA 60
Ler             ATGGGCCAAACGTTTCACGCTTTTATGTTCCCATGGTTTGCTTTTGGTCATATGACTCCA 60
Est-1*          ATGGGCCAAAATTTTCACGCTTTTATGTTCCCATGGTTTGCTTTTGGTCATATGACTCCA 60
Est-1           ATGGGCCAAACGTTTCACGCTTTTATGTTCCCATGGTTCGCTTTTGGTCATATGACTCCA 60
                **********  ************************** *********************

Col-0           TACTTGCATCTAGCCAACAAGCTAGCTGCTAAAGGTCATAGGGTTACTTTCTTGCTGCCT 120
Est             TACTTGCATCTAGCCAACAAGCTAGCTGCTAAAGGTCATAGGGTTACTTTCTTGCTGCCT 120
Kond            TACTTGCATCTAGCCAACAAGCTAGCTGCTAAAGGTCATAGGGTTACTTTCTTGCTGCCT 120
Tsu-1           TACTTGCATCTAGCCAACAAGCTAGCTGCTAAAGGTCATAGGGTTACTTTCTTGCTGCCT 120
An-1            TACTTGCATCTAGCCAACAAGCTAGCTGCTAAAGGTCATAGGGTTACTTTCTTGCTGCCT 120
Ler             TACTTGCATCTAGCCAACAAGCTAGCTGCTAAAGGTCATAGGGTTACTTTCTTGCTGCCT 120
Est-1*          TACTTGCATCTAGCCAACAAGCTAGCTGCTAAAGGTCATAGGGTTACTTTCTTGCTGCCT 120
Est-1           TACTTGCATCTAGCCAACAAGCTAGCTGCTAAAGGTCATAGGGTTACTTTCTTGCTGCCT 120
                ************************************************************

Col-0           AAGAAAGCTCAAAAACAGTTGGAACATCACAATCTGTTTCCAGACAGGATCATCTTTCAT 180
Est             AAGAAAGCTCAAAAACAGTTGGAACATCACAATCTGTTTCCAGACAGGATCATCTTTCAT 180
Kond            AAGAAAGCTCAAAAACAGTTGGAACATCACAATCTGTTTCCAGACAGGATCATCTTTCAT 180
Tsu-1           AAGAAAGCTCAAAAACAGTTGGAACATCACAATCTGTTTCCAGACAGGATCATCTTTCAT 180
An-1            AAGAAAGCTCAAAAACAGTTGGAACATCACAATCTGTTTCCAGACAGGATCATCTTTCAT 180
Ler             AAGAAAGCTCAAAAACAGTTGGAACATCACAATCTGTTTCCAGACAGGATCATCTTTCAT 180
Est-1*          AAGAAAGCTCAAAAACAGTTGGAACATCACAATCTGTTTCCAGACAGGATCATCTTTCAT 180
Est-1           AAGAAAGCTCAAAAACAGTTGGAACATCACAATCTGTTTCCAGACAGGATCATCTTTCAT 180
                ************************************************************
```

```
Col-0            TCTCTTACTATTCCCCATGTTGATGGCCTACCTGCTGGCGCGGAGACCGCCTCGGACATC 240
Est              TCTCTTACTATTCCCCATGTTGATGGCCTACCTGCTGGCGCGGAGACCGCCTCGGACATC 240
Kond             TCTCTTACTATTCCCCATGTTGATGGCCTACCTGCTGGCGCGGAGACCGCCTCGGACATC 240
Tsu-1            TCTCTTACTATTCCCCATGTTGATGGCCTACCTGCTGGCGCGGAGACCGCCTCGGACATC 240
An-1             CCTCTTACTATTCCTCATGTTGATGGCCTACCTGCTGGCGCGGAGACCGCCTCGGACATC 240
Ler              CCTCTTACTATTCCTCATGTTGATGGCCTACCTGCTGGCGCGGAGACCGCCTCGGACATC 240
Est-1*           CCTCTTACTATTCCTCATGTTGATGGCCTACCTGCTGGCGCGGAGACCGCCTCGGACATC 240
Est-1            CCTCTTACTATTCCTCATGTTGATGGCCTACCTGCTGGCGCGGAGACCGCCTCGGACATC 240
                  ************* *********************************************

Col-0            CCCATCTCGTTGGGGAAGTTTCTTACCGCAGCCATGGATCTCACTCGCGATCAGGTCGAA 300
Est              CCCATCTCGTTGGGGAAGTTTCTTACCGCAGCCATGGATCTCACTCGCGATCAGGTCGAA 300
Kond             CCCATCTCGTTGGGGAAGTTTCTTACCGCAGCCATGGATCTCACTCGCGATCAGGTCGAA 300
Tsu-1            CCCATCTCGTTGGGGAAGTTTCTTACCGCAGCCATGGATCTCACTCGCGATCAGGTCGAA 300
An-1             CCCATCTCGTTGGGGAAGTTTCTTACCGCAGCCATGGATCTCACTCGCGATCAGGTCGAA 300
Ler              CCCATCTCGTTGGGGAAGTTTCTTACCGCAGCCATGGATCTCACTCGCGATCAGGTCGAA 300
Est-1*           CCCATCTCGTTGGGGAAGTTTCTTACCGCAGCCATGGATCTCACTCGCGATCAGGTCGAA 300
Est-1            CCCATCTCGTTGGGGAAGTTTCTTACCGCAGCCATGGATCTCACTCGCGATCAGGTCGAA 300
                 ************************************************************

Col-0            GCCGCGGTTCGTGCTTTGAGACCAGACCTGATCTTTTTCGATACTGCTTATTGGGTTCCG 360
Est              GCCGCGGTTCGTGCTTTGAGACCAGACCTGATCTTTTTCGATACTGCTTATTGGGTTCCG 360
Kond             GCCGCGGTTCGTGCTTTGAGACCAGACCTGATCTTTTTCGATACTGCTTATTGGGTTCCG 360
Tsu-1            GCCGCGGTTCGTGCTTTGAGACCAGACCTGATCTTTTTCGATACTGCTTATTGGGTTCCG 360
An-1             GCCGCGGTTCGTGCTTTGCGACCAGACCTGATCTTTTTCGATACTGCTTATTGGGTTCCG 360
Ler              GCCGCGGTTCGTGCTTTGCGACCAGACCTGATCTTTTTCGATACTGCTTATTGGGTTCCG 360
Est-1*           GCCGCGGTTCGTGCTTTGCGACCAGACCTGATCTTTTTCGATACTGCTTATTGGGTTCCG 360
Est-1            GCCGCGGTTCGTGCTTTGCGACCAGACCTGATCTTTTTCGATACTGCTTATTGGGTTCCG 360
                 ****************** *****************************************
```

```
Col-0           GAAATGGCGAAAGAACACAGAGTCAAGAGTGTGATATACTTTGTGATATCAGCTAACTCC 420
Est             GAAATGGCGAAAGAACACAGAGTCAAGAGTGTGATATACTTTGTGATATCAGCTAACTCC 420
Kond            GAAATGGCGAAAGAACACAGAGTCAAGAGTGTGATATACTTTGTGATATCAGCTAACTCC 420
Tsu-1           GAAATGGCGAAAGAACACAGAGTCAAGAGTGTGATATACTTTGTGATATCAGCTAACTCC 420
An-1            GAAATGGCGAAAGAACATAGAGTCAAGAGTGTGATGTACTTCGTGATATCAGCTAACTCC 420
Ler             GAAATGGCGAAAGAACATAGAGTCAAGAGTGTGATGTACTTCGTGATATCAGCTAACTCC 420
Est-1*          GAAATGGCGAAAGAACATAGAGTCAAGAGTGTGATGTACTTCGTGATATCAGCTAACTCC 420
Est-1           GAAATGGCGAAAGAACATAGAGTCAAGAGTGTGATGTACTTCGTGATATCAGCTAACTCC 420
                ***************** ***************** ***** ******************

Col-0           ATAGCTCATGAACTTGTACCAGGTGGTGAATTAGGAGTTCCTCCACCTGGCTATCCTTCG 480
Est             ATAGCTCATGAACTTGTACCAGGTGGTGAATTAGGAGTTCCTCCACCTGGCTATCCTTCG 480
Kond            ATAGCTCATGAACTTGTACCAGGTGGTGAATTAGGAGTTCCTCCACCTGGCTATCCTTCG 480
Tsu-1           ATAGCTCATGAACTTGTACCAGGTGGTGAATTAGGAGTTCCTCCACCTGGCTATCCTTCG 480
An-1            ATAGCTCATGAACTTGTACCAGGTGGTGAATTAGGAGTTCCTCCACCTGGCTATCCTTCG 480
Ler             ATAGCTCATGAACTTGTACCAGGTGGTGAATTAGGAGTTCCTCCACCTGGCTATCCTTCG 480
Est-1*          ATAGCTCATGAACTTGTACCAGGTGGTGAATTAGGAGTTCCTCCACCTGGCTATCCTTCG 480
Est-1           ATAGCTCATGAACTTGTACCAGGTGGTGAATTAGGAGTTCCTCCACCTGGCTATCCTTCG 480
                ************************************************************

Col-0           TCAAAAGTGTTGTACCGTGGACACGATGCTCACGCTTTGTTGACTTTTTCCATCTTCTAC 540
Est             TCAAAAGTGTTGTACCGTGGACACGATGCTCACGCTTTGTTGACTTTTTCCATCTTCTAC 540
Kond            TCAAAAGTGTTGTACCGTGGACACGATGCTCACGCTTTGTTGACATTTTCCATCTTCTAC 540
Tsu-1           TCAAAAGTGTTGTACCGTGGACACGATGCTCACGCTTTGTTGACTTTTTCCATCTTCTAC 540
An-1            TCAAAAGTGTTGTACCGTGGACACGATGCTCACGCTTTGTTGACTTTTTCCATCTTCTAC 540
Ler             TCAAAAGTGTTGTACCGTGGACACGATGCTCACGCTTTGTTGACTTTTTCCATCTTCTAC 540
Est-1*          TCAAAAGTGTTGTACCGTGGACACGATGCTCACGCTTTGTTGACTTTTTCCATGTTCTAC 540
Est-1           TCAAAAGTGTTGTACCGTGGACACGATGCTCACGCTTTGTTGACTTTTTCCATCTTCTAC 540
                ******************************************** ******** ******
```

```
Col-0     GAGAGGCTTCATTACCGGATAACAACAGGTCTAAAGAATTGTGATTTTATCTCAATTAGG 600
Est       GAGAGGCTTCATTACCGGATAACAACAGGTCTAAAGAATTGTGATTTTATCTCAATTAGG 600
Kond      GAGAGGCTTCATTACCGGATAACAACAGGTCTAAAGAATTGTGATTTTATCTCAATTAGG 600
Tsu-1     GAGAGGCTTCATTACCGGATAACAACAGGTCTAAAGAATTGTGATTTTATCTCAATTAGG 600
An-1      GAGAGGCTTCATCACCGGATAACAACAGGTCTAAAGAATTGTGATTTTATCTCTATTAGG 600
Ler       GAGAGGCTTCATCACCGGATAACAACAGGTCTAAAGAATTGTGATTTTATCTCTATTAGG 600
Est-1*    GAGAGGCTTCATCACCGGATAACAACAGGTCTAAAGAATTGTGATTTTATCTCTATTAGG 600
Est-1     GAGAGGCTTCATCACCGGATAACAACAGGTCTAAAGAATTGTGATTTTATCTCTATTAGG 600
          ************ **************************************** ******

Col-0     ACTTGTAAAGAAATCGAAGGTAAATTCTGCGACTATATAGAGCGTCAATACCAGAGGAAG 660
Est       ACTTGTAAAGAAATCGAAGGTAAATTCTGCGACTATATAGAGCGTCAATACCAGAGGAAG 660
Kond      ACTTGTAAAGAAATCGAAGGTAAATTCTGCGACTATATAGAGCGTCAATACCAGAGGAAG 660
Tsu-1     ACTTGTAAAGAAATCGAAGGTAAATTCTGCGACTATATAGAGCGTCAATACCAGAGGAAG 660
An-1      ACTTGTAAAGAAGTCGAAGGTAAATTCTGCGACTATATAGAGCGTCAATACCAGAGGAAG 660
Ler       ACTTGTAAAGAAGTCGAAGGTAAATTCTGCGACTATATAGAGCGTCAATACCAGAGGAAG 660
Est-1*    ACTTGTAAAGAAGTCGAAGGTAAATTCTGCGACTATATAGAGCGTCAATACCAGAGGAAG 660
Est-1     ACTTGTAAAGAAGTCGAAGGTAAATTCTGCGACTATATAGAGCGTCAATACCAGAGGAAG 660
          ************ ***********************************************

Col-0     GTTCTTTTGACAGGTCCAATGCTTCCAGAGCCAGATAACAGTAGACCACTCGAAGATCGA 720
Est       GTTCTTTTGACAGGTCCAATGCTTCCAGAGCCAGATAACAGTAGACCACTCGAAGATCGA 720
Kond      GTTCTTTTGACAGGTCCAATGCTTCCAGAGCCAGATAACAGTAGACCACTCGAAGATCGA 720
Tsu-1     GTTCTTTTGACAGGTCCAATGCTTCCAGAGCCAGATAACAGTAGACCACTCGAAGATCGA 720
An-1      GTTCTTTTGACAGGTCCAATGCTTCCAGAGCCAGACAATAGTAGACCACTCGAAGATCGA 720
Ler       GTTCTTTTGACAGGTCCAATGCTTCCAGAGCCAGACAATAGTAGACCACTCGAAGATCGA 720
Est-1*    GTTCTTTTGACAGGTCCAATGCTTCCAGAGCCAGACAATAGTAGACCACTCGAAGATCGA 720
Est-1     GTTCTTTTGACAGGTCCAATGCTTCCAGAGCCAGACAATAGTAGACCACTCGAAGATCGA 720
          *********************************** ** *********************
```

```
Col-0        TGGAATCACTGGCTGAATCAGTTCAAACCCGGCTCGGTAATATATTGTGCATTGGGAAGT 780
Est          TGGAATCACTGGCTGAATCAGTTCAAACCCGGCTCGGTAATATATTGTGCATTGGGAAGT 780
Kond         TGGAATCACTGGCTGAATCAGTTCAAACCCGGCTCGGTAATATATTGTGCATTGGGAAGT 780
Tsu-1        TGGAATCACTGGCTGAATCAGTTCAAACCCGGCTCGGTAATATATTGTGCATTGGGAAGT 780
An-1         TGGAATCACTGGCTGAATCAGTTCAAACCCGGCTCGGTAATATATTGTGCATTGGGAAGT 780
Ler          TGGAATCACTGGCTGAATCAGTTCAAACCCGGCTCGGTAATATATTGTGCATTGGGAAGT 780
Est-1*       TGGAATCACTGGCTGAATCAGTTCAAACCCGGCTCGGTAATATATTGTGCATTGGGAAGT 780
Est-1        TGGAATCACTGGCTGAATCAGTTCAAACCCGGCTCGGTAATATATTGTGCATTGGGAAGT 780
             ************************************************************

Col-0        CAAATCACTCTAGAGAAGGATCAATTCCAAGAACTCTGTTTAGGAATGGAGCTCACTGGT 840
Est          CAAATCACTCTAGAGAAGGATCAATTCCAAGAACTCTGTTTAGGAATGGAGCTCACTGGT 840
Kond         CAAATCACTCTAGAGAAGGATCAATTCCAAGAACTCTGTTTAGGAATGGAGCTCACTGGT 840
Tsu-1        CAAATCACTCTAGAGAAGGATCAATTCCAAGAACTCTGTTTAGGAATGGAGCTCACTGGT 840
An-1         CAAATAACTCTAGAGAAGGATCAATTCCAAGAACTCTGTTTAGGAATGGAGCTTACTAGT 840
Ler          CAAATAACTCTAGAGAAGGATCAATTCCAAGAACTCTGTTTAGGAATGGAGCTTACTAGT 840
Est-1*       CAAATAACTCTAGAGAAGGATCAATTCCAAGAACTCTGTTTAGGAATGGAGCTTACTAGT 840
Est-1        CAAATAACTCTAGAGAAGGATCAATTCCAAGAACTCTGTTTAGGAATGGAGCTTACTAGT 840
             ***** *********************************************** *** **

Col-0        TTACCGTTTCTCGTAGCGGTAAAACCACCAAAAGGCGCAAAGACGATCCAAGAAGCGTTG 900
Est          TTACCGTTTCTCGTAGCGGTAAAACCACCAAAAGGCGCAAAGACGATCCAAGAAGCGTTG 900
Kond         TTACCGTTTCTCGTAGCGGTAAAACCACCAAAAGGCGCAAAGACGATCCAAGAAGCGTTG 900
Tsu-1        TTACCGTTTCTCGTAGCGGTAAAACCACCAAAAGGCGCAAAGACGATCCAAGAAGCGTTG 900
An-1         TTACCGTTTCTTGTAGCGGTAAAACCACCAAAAGGCGCAAAGACGATTCAAGAAGCGTTG 900
Ler          TTACCGTTTCTTGTAGCGGTAAAACCACCAAAAGGCGCAAAGACGATTCAAGAAGCGTTG 900
Est-1*       TTACCGTTTCTTGTAGCGGTAAAACCACCAAAAGGCGCAAAGACGATTCAAGAAGCGTTG 900
Est-1        TTACCGTTTCTTGTAGCGGTAAAACCACCAAAAGGCGCAAAGACGATTCAAGAAGCGTTG 900
             *********** *********************************** ************
```

```
Col-0     CCAGAAGGGTTTGAGGAGAGGGTGAAGAATCATGGAGTAGTTTGGGGAGAATGGGTGCAG 960
Est       CCAGAAGGGTTTGAGGAGAGGGTGAAGAATCATGGAGTAGTTTGGGGAGAATGGGTGCAG 960
Kond      CCAGAAGGGTTTGAGGAGAGGGTGAAGAATCATGGAGTAGTTTGGGGAGAATGGGTGCAG 960
Tsu-1     CCAGAAGGGTTTGAGGAGAGGGTGAAGAATCATGGAGTAGTTTGGGGAGAATGGGTGCAG 960
An-1      CCAGAAGGGTTTGAAGAGAGGGTGAAGAATCATGGAGTAGTTTGGGGAGAATGGGTGCAG 960
Ler       CCAGAAGGGTTTGAAGAGAGGGTGAAGAATCATGGAGTAGTTTGGGGAGAATGGGTGCAG 960
Est-1*    CCAGAAGGGTTTGAAGAGAGGGTGAAGAATCATGGAGTGGTTTGGGGAGAATGGGTGCAG 960
Est-1     CCAGAAGGGTTTGAGGAGAGGGTGAAGAATCATGGAGTAGTTTGGGGAGAATGGGTGCAG 960
          ************** *********************** *********************

Col-0     CAACCATTGATATTGGCTCATCCATCAGTAGGCTGCTTTGTGACCCATTGTGGGTTTGGA 1020
Est       CAACCATTGATATTGGCTCATCCATCAGTAGGCTGCTTTGTGACCCATTGTGGGTTTGGA 1020
Kond      CAACCATTGATATTGGCTCATCCATCAGTAGGCTGCTTTGTGACCCATTGTGGGTTTGGA 1020
Tsu-1     CAACCATTGATATTGGCTCATCCATCAGTAGGCTGCTTTATGACCCATTGTGGGTTTGGA 1020
An-1      CAACCATTGATATTGGCTCATCCATCGGTAGGATGCTTTGTGAATCATTGTGGGTTTGGA 1020
Ler       CAACCATTGATATTGGCTCATCCATCGGTAGGATGCTTTGTGAATCATTGTGGGTTTGGA 1020
Est-1*    CAACCATTGATATTGGCTCATCCATCGGTAGGATGCTTTGTGAATCATTGTGGGTTTGGA 1020
Est-1     CAACCATTGATATTGGCTCATCCATCGGTAGGCTGCTTTGTGAATCATTGTGGGTTTGGA 1020
          ************************** ***** ****** ***  ***************

Col-0     TCAATGTGGGAGTCTCTAGTGAGTGATTGTCAAATAGTCTTGCTTCCATATTTGTGTGAT 1080
Est       TCAATGTGGGAGTCTCTAGTGAGTGATTGTCAAATAGTCTTGCTTCCATATTTGTGTGAT 1080
Kond      TCAATGTGGGAGTCTCTAGTGAGTGATTGTCAAATAGTCTTACTTCCATATTTGAGTGAT 1080
Tsu-1     TCAATGTGGGAGTCTCTAGTGAGTGATTGTCAAATAGTCTTGCTTCCATATTTGAGTGAT 1080
An-1      TCAATGTGGGAGTCTCTAGTGAGTGATTGTCAAATAGTCTTGCTTCCATATTTGAGTGAT 1080
Ler       TCAATGTGGGAGTCTCTAGTGAGTGATTGTCAAATAGTCTTGCTTCCATATTTGAGTGAT 1080
Est-1*    TCAATGTGGGAGTCTCTAGTGAGTGATTGTCAAATAGTCTTGCTTCCATATTTGTGTGAT 1080
Est-1     TCAATGTGGGAGTCTCTAGTGAGTGATTGTCAAATAGTCTTGCTTCCATATTTGAGTGAT 1080
          ***************************************** ************ *****
```

```
Col-0           CAAATTCTCAACACTAGATTGATGAGTGAGGAACTCGAGGTTTCGGTGGAAGTGAAAAGA 1140
Est             CAAATTCTCAACACTAGATTGATGAGTGAGGAACTCGAGGTTTCGGTGGAAGTGAAAAGA 1140
Kond            CAAATTCTCAACACTAGATTGATGAGTGAGGAACTCGAGGTTTCGGTGGAAGTGAAAAGA 1140
Tsu-1           CAAATTCTCAACACTAGATTGATGAGTGAGGAACTCGAGGTTTCGGTGGAAGTGAAAAGA 1140
An-1            CAAATTCTCAACACTAGATTGATGAGTGAGGAACTCGAGGTTTCGGTGGAAGTGAAAAGA 1140
Ler             CAAATTCTCAACACTAGATTGATGAGTGAGGAACTCGAGGTTTCGGTGGAAGTGAAAAGA 1140
Est-1*          CAAATTCTCAACACTAGATTGATGAGTGAGGAACTCGAGGTTTCGGTGGAAGTGAAAAGA 1140
Est-1           CAAATTCTCAACACTAGATTGATGAGTGAGGAACTCGAGGTTTCGGTGGAAGTGAAAAGA 1140
                ************************************************************

Col-0           GAAGAAACAGGATGGTTCTCGAAAGAGAGCTTAAGTGTTGCGATCACCTCGGTGATGGAC 1200
Est             GAAGAAACAGGATGGTTCTCGAAAGAGAGCTTAAGTGTTGCGATCACCTCGGTGATGGAC 1200
Kond            GAAGAAACAGGATGGTTCTCGAAAGAGAGCTTAAGTGTTGCGATCACCTCGGTGATGGAC 1200
Tsu-1           GAAGAAACAGGATGGTTCTCGAAAGAGAGCTTAAGTGTTGCGATCACCTCGGTGATGGAC 1200
An-1            GAAGAAACAGGATGGTTCTCGAAAGAGAGCTTAAGTGTTGCGATCACCTCGGTGATGGAC 1200
Ler             GAAGAAACAGGATGGTTCTCGAAAGAGAGCTTAAGTGTTGCGATCACCTCGGTGATGGAC 1200
Est-1*          GAAGAAACAGGATGGTTCTCGAAAGAGAGCTTAAGTGTTGCGATCACCTCGGTGATGGAC 1200
Est-1           GAAGAAACAGGATGGTTCTCGAAAGAGAGCTTAAGTGTTGCGATCACCTCGGTGATGGAC 1200
                ************************************************************

Col-0           AAAGATAGTGAGTTAGGGAATCTGGTGAGGAGGAACCACGCTAAATTAAAGGAGGTTTTG 1260
Est             AAAGATAGTGAGTTAGGGAATCTGGTGAGGAGGAACCACGCTAAATTAAAGGAGGTTTTG 1260
Kond            AAAGATAGTGAGTTAGGGAATCTGGTGAGGAGGAACCACGCTAAATTAAAGGAGGTTTTG 1260
Tsu-1           AAAGATAGTGAGTTAGGGAATCTGGTGAGGAGGAACCACGCTAAATTAAAGGAGGTTTTG 1260
An-1            AAAGATAGTGAGTTAGGGAATCTGGTGAGGAGGAACCACGCTAAATTAAAGGAGGTTTTG 1260
Ler             AAAGATAGTGAGTTAGGGAATCTGGTGAGGAGGAACCACGCTAAATTAAAGGAGGTTTTG 1260
Est-1*          AAAGATAGTGAGTTAGGGAATCTGGTGAGGAGGAACCACGCTAAATTAAAGGAGGTTTTG 1260
Est-1           AAAGATAGTGAGTTAGGGAATCTGGTGAGGAGGAACCACGCTAAATTAAAGGAGGTTTTG 1260
                ************************************************************
```

```
Col-0           GTTAGTCCTGGATTATTAACCGGTTACACCGATGAATTTGTTGAAACTTTGCAGAATATA 1320
Est             GTTAGTCCTGGATTATTAACCGGTTACACCGATGAATTTGTTGAAACTTTGCAGAATATA 1320
Kond            GTTAGTCCTGGATTATTAACCGGTTACACCGATGAATTTGTTGAAACTTTGCAGAATATA 1320
Tsu-1           GTTAGTCCTGAATTATTAACCGGTTACACCGATGAATTTGTTGAAACTTTGCAGAATATA 1320
An-1            GTTAGTCCTGGATTATTAACCGGTTACACCGATGAATTTGTTGAAACTTTGCAGAATATA 1320
Ler             GTTAGTCCTGGATTATTAACCGGTTACACCGATGAATTTGTTGAAACTTTGCAGAATATA 1320
Est-1*          GTTAGTCCTGGATTATTAACCGGTTACACCGATGAATTTGTTGAAACTTTGCAGAATATA 1320
Est-1           GTTAGTCCTGGATTATTAACCGGTTACACCGATGAATTTGTTGAAACTTTGCAGAATATA 1320
                ********** *************************************************

Col-0           GTCAACGATACAAATCTTGAATGA 1344
Est             GTCAACGATACAAATCTTGAATGA 1344
Kond            GTCAACGATACAAATCTTGAATGA 1344
Tsu-1           GTCAACGATACAAATCTTGAATGA 1344
An-1            GTCAACGATACAAATCTTGAATGA 1344
Ler             GTCAACGATACAAATCTTGAATGA 1344
Est-1*          GTCAACGATACAAATCTTGAATGA 1344
Est-1           GTCAACGATACAAATCTTGAATGA 1344
                ************************
```

Figure S3. Multiple Sequence Alignment of coding sequences of *AtUDP-glycosyltransferase* gene produced by CLUSTALW. The following sequences were taken from the 1001 Genomes Project database (www.1001genomes.org): Kond, L*er*, Est, Col-0, Est-1, Tsu-1, An-1 (Kas-2 sequence was not available in the database). The selected sequences originate from accessions, which were used in the initial screening of natural variation in scopolin and scopoletin accumulation. The Est-1* sequence was obtained after re-sequencing of Est-1 accession from our laboratory.
